# Computational Model of Primary Visual Cortex Combining Visual Attention for Action Recognition

**DOI:** 10.1371/journal.pone.0130569

**Published:** 2015-07-01

**Authors:** Na Shu, Zhiyong Gao, Xiangan Chen, Haihua Liu

**Affiliations:** 1 School of Biomedical Engineering, South-Central University for Nationalities, Wuhan 430074, China; 2 Key Laboratory of Cognitive Science of State Ethnic Affairs Commission, South-Central University for Nationalities, Wuhan 430074, China; Centre de Neuroscience Cognitive, FRANCE

## Abstract

Humans can easily understand other people’s actions through visual systems, while computers cannot. Therefore, a new bio-inspired computational model is proposed in this paper aiming for automatic action recognition. The model focuses on dynamic properties of neurons and neural networks in the primary visual cortex (V1), and simulates the procedure of information processing in V1, which consists of visual perception, visual attention and representation of human action. In our model, a family of the three-dimensional spatial-temporal correlative Gabor filters is used to model the dynamic properties of the classical receptive field of V1 simple cell tuned to different speeds and orientations in time for detection of spatiotemporal information from video sequences. Based on the inhibitory effect of stimuli outside the classical receptive field caused by lateral connections of spiking neuron networks in V1, we propose surround suppressive operator to further process spatiotemporal information. Visual attention model based on perceptual grouping is integrated into our model to filter and group different regions. Moreover, in order to represent the human action, we consider the characteristic of the neural code: mean motion map based on analysis of spike trains generated by spiking neurons. The experimental evaluation on some publicly available action datasets and comparison with the state-of-the-art approaches demonstrate the superior performance of the proposed model.

## Introduction

It is a universally accepted fact that human can easily recognize and understand other peoples action from complex natural scene. It attributes the success to hundreds or thousands of neurons in visual cortex of the brain and neural networks formed by their connection in a certain way, which perceive and process motion information of human action for action recognition task. The question is how neurons and neural networks process motion information to perform this task. Researchers have made many neurophysiological studies and obtained some important findings to answer these problems. For example, the visual information is processed through two distinct pathways: the dorsal stream and the ventral stream, originating from primary visual cortex (V1). The majority of neurons in V1 are exquisitely sensitive to the orientation of a stimulus in a given position of the visual field, and their responses to a stimulus presented in the classical receptive field (RF) are often suppressed by another stimulus simultaneously presented outside the classical RF, known as “surround suppression” [1]. Based on these properties of neurons and neural mechanisms, some biophysically-plausible computational models for biological motion recognition are developed [2]. These models essentially reproduce certain properties of visual systems and make predictions for neuroscience, but have been relatively fewer reports on practical applications for human action recognition.

With the remarkable advances in the understanding of human action perception in psychophysics [3], many bio-inspired approaches of human action recognition [4]–[5] are proposed. Most of them are based on the work of M. Giese and T. Poggio [2], which puts forward a biologically plausible neural model separately to evaluate both visual pathways in biological motion recognition. These approaches are built with feedforward architecture and by modeling neural mechanism in intermediate and higher visual areas of the dorsal stream such as middle temporal (MT) and lateral medial superior temporal (MST). However, these approaches largely ignore some properties of neurons in V1 as a beginning area of visual cortex, such as inseparable properties of the classical RF of many simple cells in space and time. It hampers the processing of the shape information addressed in ventral stream and the analysis of motion information involved in dorsal stream.

Moreover, biological motion recognition can be realized in the human visual cortex with latencies of about 150ms and even faster [6], which, considering the visual pathway latencies, may only be compatible with a very specific processing architecture and mechanism. There is a neural computational theory support this mechanism, which pattern motion is computed in V1 where end-stopped cells could be involved in encoding pattern motion because they respond well to line terminators (or features) moving in their preferred direction and speed [7], [8]. The network models incorporated with feedback mechanisms have also been proposed to support the idea that pattern motion can be computed at the V1 stage [9]. In computer vision, Kornprobst [10] demonstrated that early visual processes in V1 could be sufficient to perform such task of human action recognition. Although computation of pattern motion is dynamical over space and time and is limited in V1 to reduce computation load, it does not achieve the better performance of human action recognition since many important properties of cells in V1 are not considered. Thus, it still need further research of bio-inspired approaches for human action recognition based on the properties of cells in V1.

In this paper, a new bio-inspired model is proposed for real video analysis and recognition of human actions. It focuses on three parts: 1) perceiving the spatiotemporal information by modeling properties of cells in V1 such as spatiotemporal properties of classical receptive field (RF) and surround suppression; 2) automatically detecting and localizing moving object (human) in the scene with visual attention built by the spatiotemporal information, and 3) encoding spike trains automatically generated by spiking neurons for action recognition.

According to RF properties of single neuron in V1, there are three basic RF types [11]: oriented RFs, non-oriented RFs, and non-oriented large field. In general, cells with oriented RFs are broadly modeled with filter bands to detect information in a direction from images or videos, such as 2D Gabor bands in [12] and spatiotemporal filters in [13], whereas cells with non-oriented RFs are not considered to do for it, but, by most accounts, respond optimally to moving stimuli over a restricted range of velocities. Furthermore, for a majority of cells, the spatial structure of the RF changes as a function of time can be characterized in the space-time domain [14]. These properties facilitates the detection of spatiotemporal information in different directions and at different speeds.

In addition, neurophysiological studies have also shown that the responses of neurons in V1 are suppressed by stimuli provided by the region surrounding the RF [1]. It is known as surround suppression, which is an useful mechanism for contour detection by inhibition of texture [15]. A similar mechanism has been observed in the spatiotemporal domain, where the response of such a neuron is suppressed when moving stimuli are presented in the region surrounding its classical RF. The suppression is maximal when the surround stimuli move in the same direction and at the same disparity as the preferred center stimulus [8]. An important utility of surround mechanisms in the spatiotemporal domain is to evaluate detection of motion discontinuities or motion boundaries.

To recognize human actions from clustered visual field where there are multiple moving objects, we need to automatically detect and localize every one in the actual application. Visual attention is one of the most important mechanisms of the human visual system. It can filter out redundant visual information and detect the most salient parts in our visual field. Some research works [16], [17] have shown that the visual attention is extremely helpful to action recognition. Many computational models of visual attention are raised. For example, a neurally plausible architecture is proposed by Koch and Ullman [18]. The method is highly sensitive to spatial features such as edges, shape and color, while insentitive to motion features. Although the models proposed in [17] and [19] have regarded motion features as an additional conspicuity channel, they only identify the most salient location in the sequence image but have not notion of the extent of the attended object at this location. The facilitative interaction between neurons in V1 reported in numerous studies is one of mechanisms to group and bind visual features to organize a meaningful higher-level structure [20]. It is beneficial to detect moving object.

To sum up, our goal is to build a bio-inspired model for human action recognition. In our model, spatiotemporal information of human action is detected by using the properties of neurons only in V1 without MT, moving objects are localized by simulating the visual attention mechanism based on spatiotemporal information, and actions are represented by mean firing rates of spike neurons. The remainder of this paper is organized as follows: firstly, a review of research in the area of action recognition is described. Secondly, we introduce the detection of spatiotemporal information with 3D Gabor spatial-temporal filters modeling the properties of V1 cells and their center surround interactions, and detail computational model of visual attention and the approach for human action localization. Thirdly, the spiking neural model to simulate spike neuron is adopted to transfer spatiotemporal information to spike train, and mean motion maps as feature sets of human action are employed to represent and classify human action. Finally, we present the experimental results, being compared with the earlier introduced approaches.

## Related Work

For human action recognition, the typical process includes feature extraction from image sequences, image representation and action classification. Based on image representation, the action recognition approaches can be divided into two categories [21], i.e. global or local. Both of them have achieved success for human action recognition to some extent, yet there are still some problems to be resolved. For example, the global approaches are sensitive to noise, partial occlusions and variations [22], [23], while the local ones sometimes suffer from heavy computational burden [24], [25] for extracting a sufficient amount of relevant interest points [26]. In recent years, some approaches combine both global and local representations to improve recognizing performance [27–29]. However, they are mainly applied into some special situations. Thus, some bio-inspired approaches emerge to perform the task of action recognition.

The work of bio-inspired action recognition based on the feedward architecture of visual cortex is related to several domains including motion-based recognition and local feature detection. In the area of local feature detection, a large number of different schemes have been developed based on visual properties and feature descriptors [4], [30], [31], [32]. In [4], a feedforward architecture modeling *dorsal* visual pathway was proposed by Jhuang, which can be seen as an extension of model of *ventral* pathway architecture [12] according to similar organization of both *ventral* and *dorsal* pathways [33]. Jhuang mapped the cortical architecture, essentially primary visual cortex (V1) (with simple and complex cells), but never claim any biological relevance for the corresponding subsequent processing stages (from S2 to C3) [13]. The work in [31] is similar to Jhuang’s idea in concept, but uses different window settings. Schindler and Van Gool [30] extend Jhuang’s approach [4] by combining both shape and motion responses. Due to a collection of independent features obtained in matching stage, the approach is suffering from heavy computation.

Researchers also have developed a large number of different schemes based on various combinations of visual tasks and image descriptors [5, 13]. Escobar et al. [13] still used feedforward architecture and simulated *dorsal* visual pathway to create a computational model for human action recognition, called V1-MT model, in which the analysis of motion information is done in V1 and MT areas [33]. The model not only combines motion-sensitive responses but also considers connections between V1 cells and MT cells found in [34], [35], which allows them to model more complex properties such as motion contrasts. The main difference from Jhuang’s approach is that the approach is based on Casile and Giese theory [36], which augment that biological motion recognition can be done in a coarse spatial location of the mid-level optic flow features. The visual observation of human action is encoded as a whole with spiking neural networks in [13], [5], and is considered as global representations. Although Escobar’s approach satisfies biology plausibility, there are some key problems to be solved. For example, which properties of the cells in V1 should be used to detect spatiotemporal information? how are human actions detected and localized? and how is such task of human action recognition performed through early visual processing in V1? Therefore, we aim to give some schemes to settle these issues.

## Visual Perception and Information Detection

**Fig 1:**
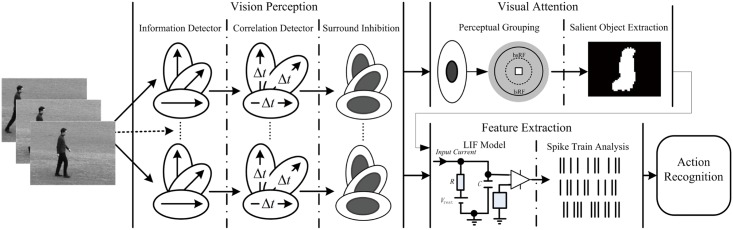
The architecture of the proposed model of visual primary cortex combining visual attention. It is consisted of four parts: visual perception, visual attention, feature extraction and action recognition. Spatiotemporal information is detected by modeling properties of classical and nonclassical receptive field of cells in V1; motion objects are detected with attention computational model by grouping spatiotemporal information; spike trains of spiking neurons produced by stimulus-driven leaky integrate-and-fire are analyzed to extract action features; the mean motion map as feature sets is constructed for action recognition with SVM classifier.

Biological visual system is very complex. Physiological and psychological studies suggest four crucial properties of biological vision: Fovea-periphery distinction on the retina, oculomotor, image representation and serial processing [37]. In this paper, we propose a novel bio-inspired approach for human action recognition according to these properties. Fig 1 shows the block diagram of our approach from the input image sequence containing human action as stimulus to its final classification. It contains four steps: 1) detecting spatiotemporal information in form of responses of simple and complex cell in V1; 2) localizing moving object with computational model of visual attention by integrating spatiotemporal information sensitive to speed and direction; 3) extracting features from spiking trains generated by spiking neurons with leaky integrate-and-fire model [38], [39], and encoding them for action representation, 4) recognizing human action with the support vector machine (SVM).

### 1 Spatiotemporal Information Detection

In V1, many simple cells possess the property of the speed and direction selectivity (oriented-cell), and their RF profiles are essentially modeled with spatiotemporal filters. However, most of existing spatiotemporal filters often are non-causal, hence biologically implausible [4, 31]. To this end, we build a family of spatiotemporal filters to model the spatiotemporal RF profiles of simple cells similar to [40], denoted by *g*_*v*,*θ*,*φ*_(**x**, *t*), which is causal and consistent with the V1 cell physiology. The formula of spatiotemporal filter is defined in Eq (1).

$$\begin{array}{cc} g_{v,\theta ,\varphi }\left(x,t\right)= & \exp\left[-\frac{{\left(\bar{x}+vt\right)}^{2}}{2\sigma^{2}}-\frac{\gamma^{2}{\bar{y}}^{2}}{2\sigma^{2}}-\frac{{\left(t-u_{t}\right)}^{2}}{2\tau^{2}}\right]\\ & •\frac{\gamma }{{\left(2\pi \right)}^{3/2}\sigma^{2}\tau }\cos\left(\frac{2\pi }{\lambda }\left(\bar{x}+vt\right)+\varphi \right) \end{array}$$ (1)

where $\left(\bar{x},\bar{y})=(x\;\text{cos}\;\theta +y\;\text{sin}\;\theta ,-xsin\theta +y\;\text{cos}\;\theta \right)$, *ɛ*(*t*) is step function, and **x** = (*x*, *y*). The parameters *v*, *θ* and *φ* respectively present the preferred speed, the preferred direction of motion and the preferred spatial orientation, and the spatial symmetry of the filter. This filter is composed of spatial Gaussian envelope and temporal Gaussian envelope. The spatiotemporal RF profile is tilted to preferred direction of motion in space-time, originating the selectivity for moving stimuli, and is qualitatively similar to the experimentally determined ones by DeAngelis [14]. Considering the correlation between preferred spatial scale and preferred speed of spatiotemporal RF profile, we use the following equation to describe the relation between the preferred spatial wavelength *λ* and the preferred speed *v*:

$$\begin{array}{c} \lambda =\lambda_{0}\sqrt{1+v^{2}} \end{array}$$ (2)

where the constant *λ*_0_ is the spatiotemporal period of the filter, *σ*/*λ* = 0.56. So, *v* determines the preferred wavelength and the receptive field size. The faster the filter speed *v* is, the larger the receptive field will be. Moreover, *τ* in the temporal Gaussian envelope, set as constant of 2.75 in [40], determines the temporal decay of *g*_*v*,*θ*,*φ*_(**x**, *t*) in time *t*. However, the temporal decay is dynamic and a function of the speed. It causes different time correlation in different preferred speeds. We therefore compute *τ* using the following function:

$$\begin{array}{c} \tau =-0.13v+2.73 \end{array}$$ (3)

A gray-scale image sequence, *I*(**x**, *t*), is first analyzed by 3D Gabor filters corresponding to the simple cells in V1. The response *r*_*v*,*θ*,*φ*_(**x**, *t*) to image sequence is computed by convolution:

$$\begin{array}{c} r_{v,\theta ,\varphi }\left(x,t\right)={|I\left(x,t\right)*g_{v,\theta ,\varphi }\left(x,t\right)|}^{+} \end{array}$$ (4)

where ∣ ⋅ ∣^+^ is an operator with half-wave rectification. From Eq (4), the response of the filer is phase sensitive. A phase insensitive response as the one of a complex cell, called Gabor energy, can be obtained by quadrature pair summation of the responses of two filters with a phase difference of *π*/2 as follows:

$$\begin{array}{c} {\bar{r}}_{v,\theta }\left(x,t\right)=\sqrt{r_{v,\theta ,0}^{2}\left(x,t\right)+r_{v,\theta ,\pi /2}^{2}\left(x,t\right)} \end{array}$$ (5)

**Fig 2:**
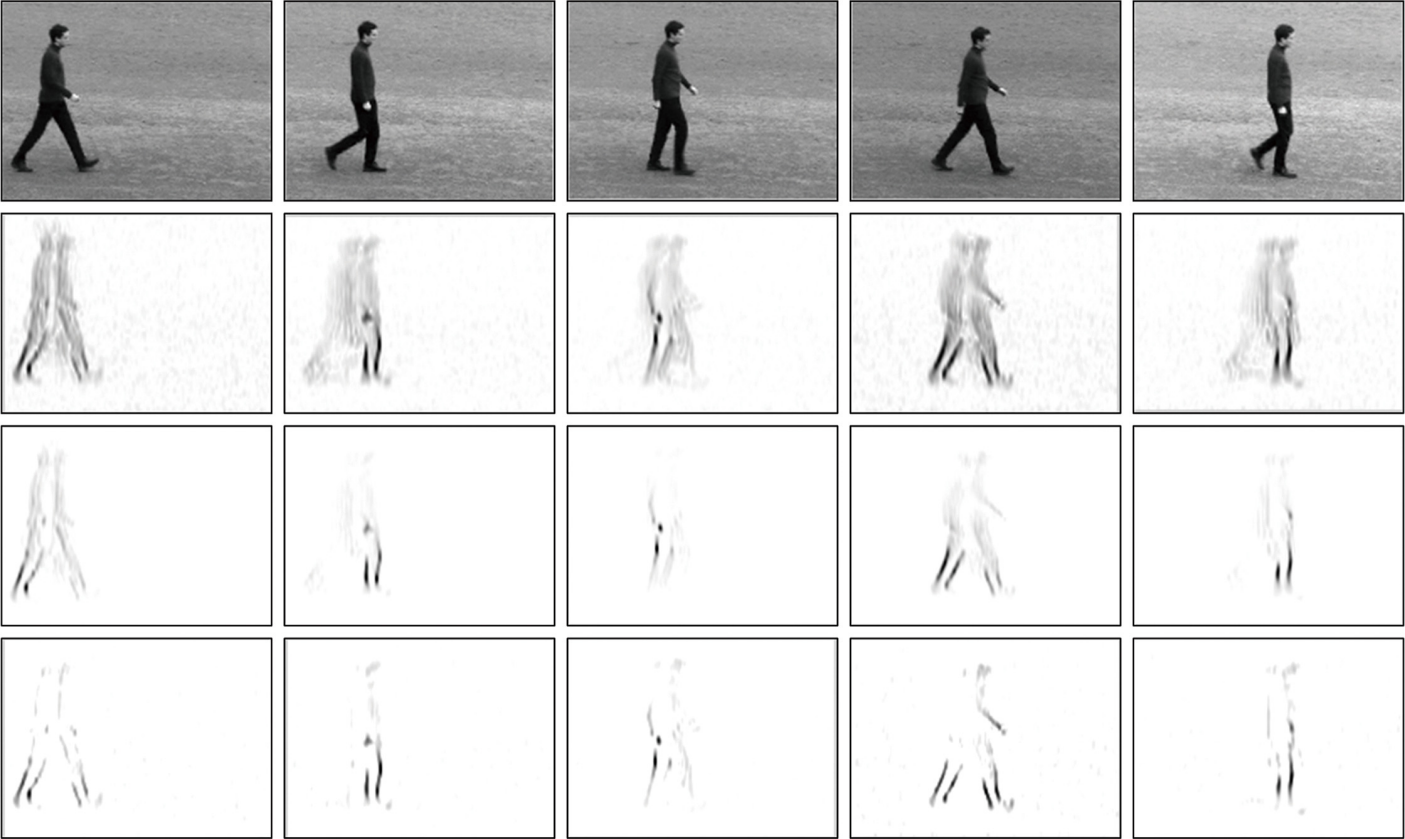
Motion information detection. *First row* shows the snapshots from a video sequence in KTH database. *Second row* shows the Gabor energy with 3D Gabor filter in 0° orientation at 1*ppF* speed. Motion energy with correlation detection is shown in the *third row* and the *fourth row* is surround suppression motion energy of *third row*. (reverse from 2*nd* to 4*th* row).

In form of Eq (5), the application for detection of spatiotemporal information is illustrated in Fig 2 (*Second Row*).

Besides oriented cells in V1, there are also some insensitive simple cells to direction (non-oriented cell). Watson et al. [41] suggested a causal temporal filter for non-oriented cell, which is consistent with the electrophysiological studies and the psychophysical data. The speed tuning properties are also studied by considering the responses of motion energy filters to motion stimulus at different speeds without orientation selectivity. For the sake of computation, however, the response of non-oriented cell is approximatively computed with Gabor energy in all directions:

$$\begin{array}{c} {\bar{r}}_{v}\left(x,t\right)=\frac{1}{N_{\theta }}\sum_{\theta }{\bar{r}}_{v,\theta }\left(x,t\right) \end{array}$$ (6)

where *N*_*θ*_ is number of preferred orientations.

As spatiotemporal information for a specific range of speeds at each location **x**, local Gabor energy, detected in Eqs (5) and (6), often is ambiguous [9]. To stabilize and disambiguate initial spatiotemporal information, a modified detector defined by a shift ∂**x** = (∂*x*, ∂*y*) along a specific speed between two successive frames is used to model complex cells to compute a spatiotemporal correlation. Similar to [9], unambiguous or disambiguated motion information is computed as following:

$$\begin{array}{c} {\hat{r}}_{v,\theta }\left(x,t\right)={\bar{r}}_{v,\theta }\left(x+\partial x,t-1\right)\cdot {\bar{r}}_{v,\theta }\left(x,t\right) \end{array}$$ (7)

$$\begin{array}{c} {\hat{r}}_{v}\left(x,t\right)={\bar{r}}_{v}\left(x,t-1\right)\cdot {\bar{r}}_{v}\left(x,t\right) \end{array}$$ (8)

The resulting activities ${\hat{r}}_{v,(\theta )}(\text{x},t)$ of different directions (including non-direction) at different speeds indicate unambiguous motion at corners and line endings, ambiguous motion along contrasts and no motion for homogeneous regions, as shown in Fig 2 (*Third Row*).

To characterize the motion in video scene, we compute the motion energy using 3D Gabor filters with *N*_*v*_ different speeds and *N*_*o*_ different directions. At each speed *v*, *N*_*o*_ + 1 responses in *N*_*o*_ directions and one non-direction are computed.

### 2 Center Surround Interaction

To further process motion information, center surround interactions are used. Surround interactions observed in V1 [1] originate from horizontal interconnections between neurons in spiking neural networks according to results of some anatomic studies, which often are antagonistic for RFs of many cells in V1. The response of such a neuron is suppressed when moving stimuli are presented in the region surrounding its classical RF.

In the purely spatial domain, a model with a 2D difference of Gaussian (DoG) functions is used to compute the spatial summation properties of a center-surround cell [42]. In spatio-temporal domain, due to RF dynamics, we define the surround suppression weighting function $w_{v,\theta }^{\left(k_{1},k_{2}\right)}$ with the half-wave-rectified difference of two concentric Gaussian envelopes:

$$\begin{array}{c} w_{v,\theta }^{\left(k_{1},k_{2}\right)}\left(x,t\right)=\frac{{|G_{v,k_{1},\theta }\left(x,t\right)-G_{v,k_{2},\theta }\left(x,t\right)|}^{+}}{{\|{|G_{v,k_{1},\theta }\left(x,t\right)-G_{v,k_{2},\theta }\left(x,t\right)|}^{+}\|}_{1}} \end{array}$$ (9)

where ‖ ⋅ ‖_1_ denotes the *L*_1_ norm and *G*_*v*,*k*,*θ*_(**x**, *t*) is similar to RF function *g*_*v*,*θ*,*φ*_(**x**, *t*), but without the cosine factor, decaying with time:

$$\begin{array}{cc} G_{v,k,\theta }\left(x,t\right)= & \frac{\gamma }{2\pi {\left(k\sigma \right)}^{2}}\text{exp}\;\left[-\frac{{\left(\bar{x}+vt\right)}^{2}+\gamma^{2}{\bar{y}}^{2}}{2{\left(k\sigma \right)}^{2}}\right]\\ & •\frac{1}{\sqrt{2\pi \tau }}\text{exp}\;\left[-\frac{{\left(t-u_{t}\right)}^{2}}{2\tau^{2}}\right]\;\varepsilon (t) \end{array}$$ (10)

Moreover, the non-oriented cells also show characteristic of center surround [43]. Therefore, the non-oriented term *G*_*v*,*k*_(**x**, *t*) is similarly defined as follows:

$$\begin{array}{cc} G_{v,k}\left(x,t\right)= & \frac{1}{2\pi {\left(k\sigma^{\prime }\right)}^{2}}\text{exp}\;\left[-\frac{x^{2}+y^{2}}{2{\left(k\sigma^{\prime }\right)}^{2}}\right]\\ & •\frac{1}{\sqrt{2\pi \tau }}\text{exp}\;\left[-\frac{{\left(t-u_{t}\right)}^{2}}{2\tau^{2}}\right]\;\varepsilon \left(t\right) \end{array}$$ (11)

where *σ*′ = *σ* + 0.05*σt*. To be consistent with the surround effect, the value of the surround weighting function should be zero inside the RF, and be positive outside it but dissipate with distance. Therefore, we set *k*_2_ = 1 and *k*_1_ = *k*, *k* > 1. In order to facilitate the description of oriented and non-oriented terms, we use $w_{v,(\theta )}^{\left(k\right)}(\text{x},t)$ to denote $w_{v,\theta }^{\left(k_{1},k_{2}\right)}(\text{x},t)$ and $w_{v}^{\left(k_{1},k_{2}\right)}(\text{x},t)$.

**Fig 3:**
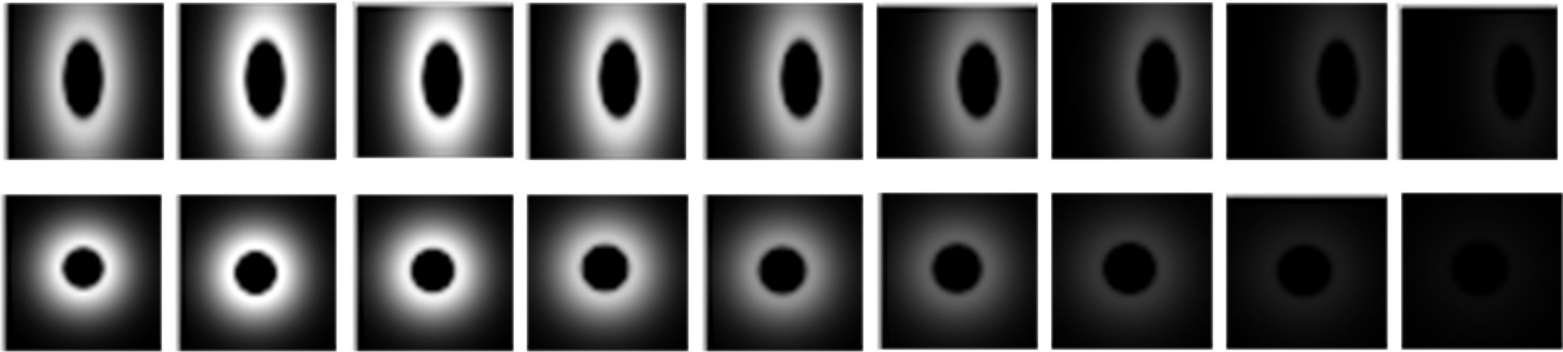
Spatiotemporal behavior of the corresponding oriented and non-oriented surround weighting function. The first row contains the profile of oriented weighting function *w*_*v*,*θ*_(**x**, *t*) with *v* = 1*ppF* and *θ* = 0, and the second row contains the profile of non-oriented weighting function *w*_*v*_(**x**, *t*) with *v* = 1*ppF*

Thus, for each point in the (**x**, *t*) space, we compute a surround suppressive motion energy $R_{v,(\theta )}^{\left(k\right)}(\text{x},t)$ as follows:

$$\begin{array}{c} R_{v,(\theta )}^{\left(k\right)}\left(x,t\right)={|{\hat{r}}_{v,(\theta )}\left(x,t\right)-\alpha {\hat{r}}_{v,(\theta )}\left(x,t\right)*w_{v,(\theta )}^{\left(k\right)}\left(x,t\right)|}^{+} \end{array}$$ (12)

where the factor *α* controls the strength with which surround suppression is taken into account. The proposed inhibition scheme is a subtractive linear mechanism followed by a nonlinear half-wave rectification (results shown in Fig 2 (*Fourth Row*)). The inhibitory gain factor *α* is unitless and represents the transformation from excitatory current to inhibitory current in the excitatory cell. It is seen that the larger and denser the motion energy ${\hat{r}}_{v,(\theta )}(\text{x},t)$ in the surroundings of a point (**x**, *t*) is, the larger the center surround term ${\hat{r}}_{v,(\theta )}(\text{x},t)*w_{v,(\theta )}^{\left(k\right)}(\text{x},t)$ is at that point. The suppression will be strongest when the stimuli in the surroundings of a point have the same direction and speed of movement as the stimulus in the concerned point. Fig 3 shows spatiotemporal behavior of the corresponding oriented and non-oriented center surround weighting function.

## Attention Model and Object Localization

**Fig 4:**
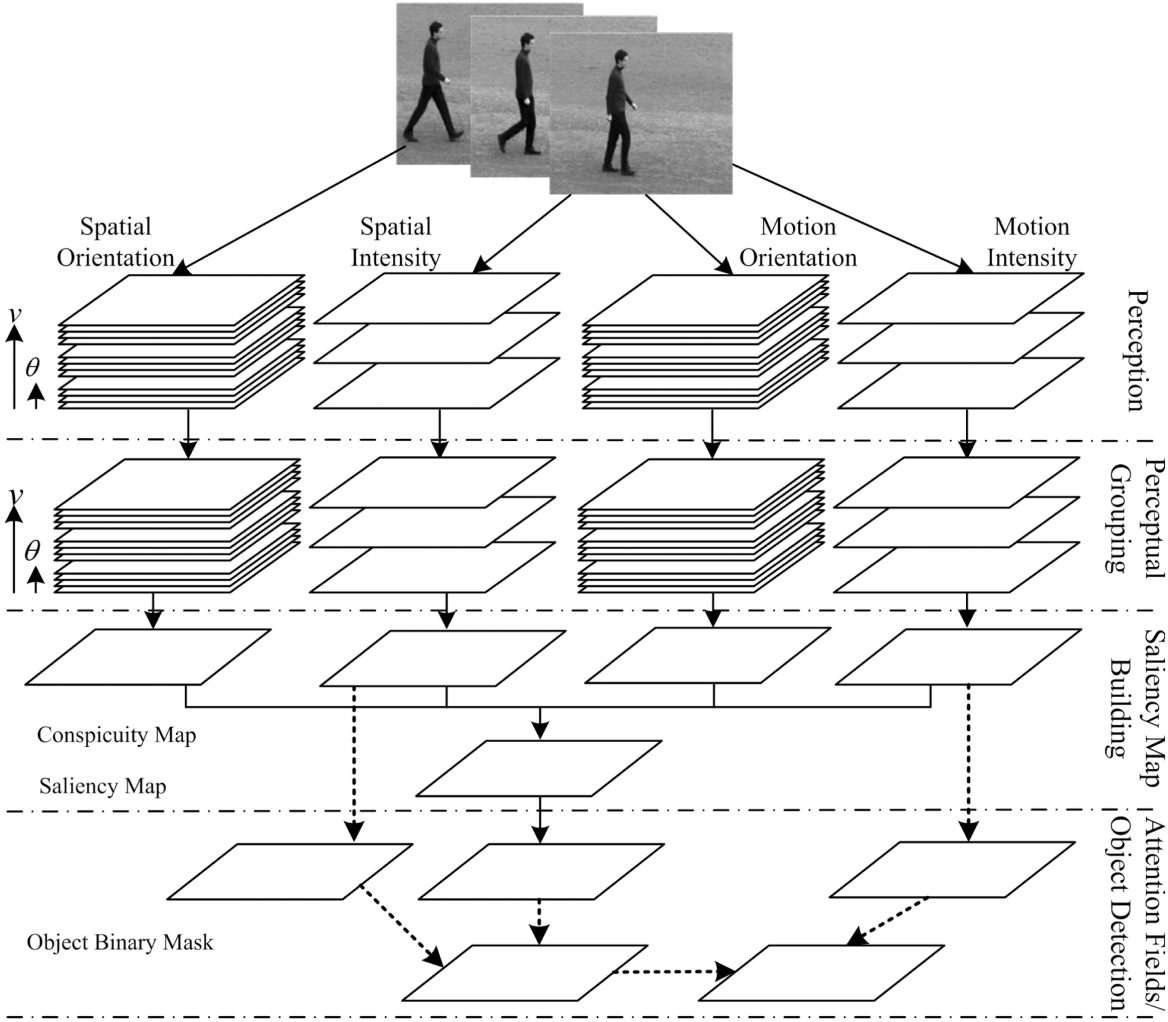
Flow chart of the proposed computational model of bottom-up visual selective attention. It presents four aspects of the vision: perception, perceptual grouping, saliency map building and attention fields. The perception is to detect visual information and suppress the redundant by simulating the behavior of cortical cells. Perceptual grouping is used to build integrative feature maps. Saliency map building is used to fuse feature maps to obtain saliency map. Finally, attention fields are achieved from saliency map.

Visual attention can enhance object localization and identification in a cluttering environment by giving more attention to salient locations and less attention to unimportant regions. Thus, Itti and Koch have proposed an attention computational model efficiently computing a saliency map from a given picture [44] based on the work of Koch and Ullman [18]. Although some models [17] and [19] try to introduce motion features into Itti’s model for moving object detection, these models have no notion of the extent of the salient moving object region. Therefore, we propose a novel attention model to localize the moving objects. Fig 4 graphically illustrates the visual attention model. The model is consistent with four steps of visual information processing, i.e. perception, perceptual grouping, saliency map building and attention fields.

In the proposed model, visual perception is implemented by spatiotemporal information detection in above section. Because we only consider gray video sequence, visual information is divided into two classes: intensity information and orientation information, which are processed in both time (motion) and space domains respectively, forming four processing channels. Each type of the information is calculated with the similar method in corresponding temporal and spatial channels, but spatial features are computed with perceiving information at low preferred speeds no more than 1*ppF*. The conspicuity maps can be re-used to obtain motion object mask instead of only using the saliency map.

### 1 Perceptual Grouping

In general, the distribution of visual information perceived generally is scattered in space (as shown in Fig 2). To organize a meaningful higher-level object structure, we should refer to human visual ability to group and bind visual information by perceptual grouping. The perceptual grouping involves numerous mechanisms. Some of computational models about perceptual grouping are based on the Gestalt principles of colinearity and proximity [45]. Others are based on surround interaction of horizontal interconnections between neurons [46], [47].

**Fig 5:**
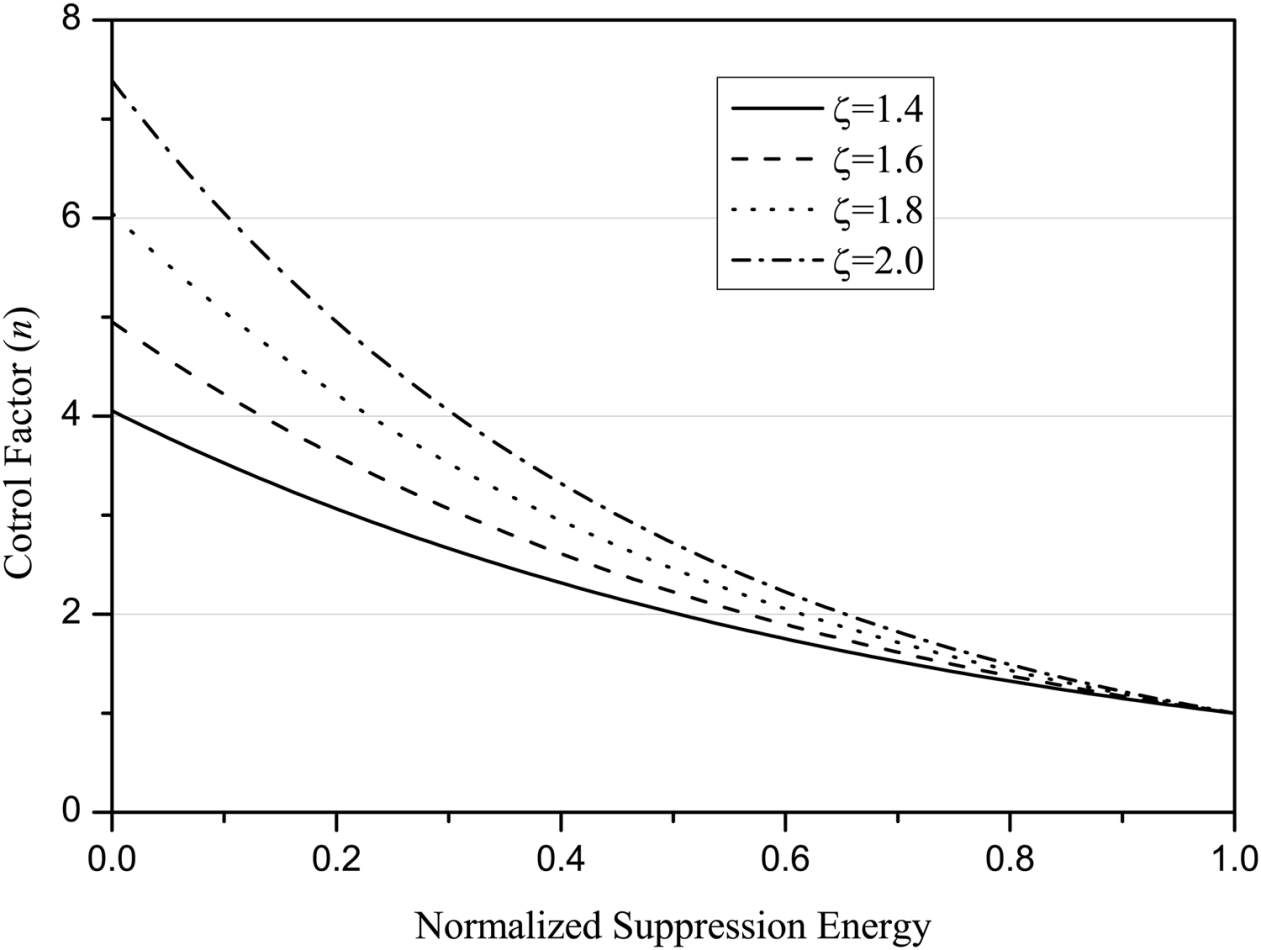
The control factor of standard deviations of the Gaussian envelopes as a function of normalized surround suppression motion energy used to compute range of perceptual grouping and weight facilitative interaction.

Besides antagonistic surround described in above section, neurons with facilitative surround structures have also been found [1], and they show an increased response when motion is presented to their surround. This facilitative interaction is always simulated using a butterfly filter [46]. In order to make the best use of dynamic properties of neurons in V1 and simplify computational architecture, we still use surround weighting function $w_{v,(\theta )}^{\left(k\right)}(\text{x},t)$ defined in Eq (9) to compute the facilitative weight, but the value of *θ* is repaced by *θ* + *π*/2. For each location (**x**, *t*) in oriented and non-oriented subbands {*v*,(*θ*)}, the facilitative weight is computed as follows:

$$\begin{array}{c} h_{v,(\theta )}^{\left(k\right)}\left(x,t\right)=R_{v,(\theta )}^{\left(k\right)}*\;w_{v,(\theta )}^{\left(n\right)} \end{array}$$ (13)

where *n* is the control factor for size of the surrounding area. According to the studies of neuroscience, the evidence shows that the spatial interactions depend crucially on the contrast, thereby allowing the visual system to register motion information efficiently and adaptively [48]. That is to say, the interactions differ for low- and high-contrast stimuli: facilitation mainly happens at low contrast and suppression occurs at high contrast [49]. They also exhibit contrast-dependent size-tuning, with lower contrasts yielding larger sizes [50]. Therefore, The spatial surrounding area determined by *n* in Eq (13) dynamically depends on the contrast of stimuli. In a certain sense, $R_{v,(\theta )}^{\left(k\right)}$ presents the contrast of motion stimuli in video sequence. Therefore, according to neurophysiological data [48], *n* is the function of $R_{v,(\theta )}^{\left(k\right)}$, defined as follows:

$$\begin{array}{c} n\left(x,t\right)=\text{exp}\;[\zeta \left(1-R_{v,(\theta )}^{\left(k\right)}\left(x,t\right)\right)] \end{array}$$ (14)

where *ζ* is a constant and not more than 2, $R_{v,(\theta )}^{\left(n\right)}(\text{x},t)$ is normalized. The *n*(**x**, *t*) function is plotted in Fig 5. For computation and performance sake, set *ζ* = 1.6 according to Fig 5 and round down *n*(**x**, *t*), *n* = ⌊*n*(**x**, *t*)⌋.

Similar to [46], the facilitative subband $O_{v,(\theta )}^{\left(k\right)}(\text{x},t)$ is obtained by weighting the subband $R_{v,(\theta )}^{\left(k\right)}$ by a factor *κ*(**x**, *t*) depending on the ratio of the local maximum of the facilitative weight $h_{v,(\theta )}^{\left(k\right)}(\text{x},t)$ and on the global maximum of this weight computed on all subbands. The resulting subband is thus given by

$$\begin{array}{c} O_{v,(\theta )}^{k}\left(x,t\right)=R_{v,(\theta )}^{\left(k\right)}\left(x,t\right)+\kappa \left(x,t\right)h_{v,(\theta )}^{\left(k\right)}\left(x,t\right) \end{array}$$ (15)

with

$$\begin{array}{c} \kappa \left(x,t\right)=\frac{\max_{x}h_{v,\theta }^{\left(k\right)}\left(x,t\right)}{\max_{\left(\cdot \right)}\left[\max_{x}h_{v,\theta }^{\left(k\right)}\left(x,t\right)\right]} \end{array}$$ (16)

where (⋅) is *θ* for oriented subband and *v* for non-oriented subband.

### 2 Saliency Map Building

To integrate all spatiotemporal information, similar to Itti’s model [44], we calculate a set of the intensity (non-orientd) feature maps 𝓕_*v*_(**x**, *t*) in terms of each feature dimension as follows:

$$\begin{array}{c} {𝓕}_{v}\left(x,t\right)={⊕}_{k}\left(O_{v}^{\left(k\right)}\left(x,t\right)\right) \end{array}$$ (17)

where we set *k* ∈ {2, 3, 4} in term $O_{v}^{\left(k\right)}(\text{x},t)$, and ⊕ is point-by-point plus operation through across-scale addition.

Another set of the orientation feature maps also are computed by similar method as follows:

$$\begin{array}{c} {𝓕}_{v,\theta }\left(x,t\right)={⊕}_{k}\left(O_{v,\theta }^{\left(k\right)}\left(x,t\right)\right) \end{array}$$ (18)

Each set of feature maps computed are divided into two classes in according to speeds. One class includes spatial feature maps obtained at speeds no more than 1*ppF*, and another class contains the motion feature maps. To guide the selection of attended locations, different feature maps need to be combined. The feature maps are then combined into four conspicuity maps: spatial orientation *F*_*o*_ and intensity *F*; motion orientation *M*_*o*_ and intensity *M*:

$$\begin{array}{c} F=\sum_{v\leq 1}{𝓕}_{v}\left(x,t\right)\;\text{and}\;M=\sum_{v>1}{𝓕}_{v}\left(x,t\right) \end{array}$$ (19)

$$\begin{array}{c} F_{o}=\sum_{v\leq 1}\sum_{\theta }{𝓕}_{v,\theta }\left(x,t\right)\;\text{and}\;M_{o}=\sum_{v>1}\sum_{\theta }{𝓕}_{v,\theta }\left(x,t\right) \end{array}$$ (20)

Because modalities of the four separative maps above contribute independently to the saliency map, we need integrate them together. Due to different dynamic ranges and extraction mechanisms, a map normalization operator, 𝓝(⋅), is globally employed to promote maps. The four conspicuity maps are then normalized and summed into the saliency map (SM) *S*:

$$\begin{array}{c} S=𝓝\left(F_{o}\right)+𝓝\left(F\right)+𝓝\left(M_{o}\right)+𝓝\left(M\right) \end{array}$$ (21)

### 3 Salient Object Extraction

Although the saliency map *S* defines the most salient location in image, to which the attentional focus should be directed, at any given time, it does not give the regions of suspicious objects. Thus, some methods with adaptive threshold [51] are proposed to obtain a binary mask (BM) of the suspicious objects from the saliency map. However, these methods only are suitable for simple still images, but not for the complex video. Therefore, we propose a sampling method to enhance BM. Let a window *W* slide on the saliency map, then sum up the values of all pixels in the window as the ‘salient degree’ of the window, defined as follows:

$$\begin{array}{c} S_{W}=\sum_{x\in W}S\left(x,t\right) \end{array}$$ (22)

where *S*(**x**, *t*) represents the saliency value of the pixel at position **x**. The size of *W* is determined by the RF size in our experiments. Consequently, we obtain *r* salient degree values *S*_*W*_*i*__, *i* = 1, ⋯, *r*. Similar to [51], the adaptive threshold (*Th*) value is regarded as the mean value of a given salient degree:

$$\begin{array}{c} Th=k\sum_{i=1}^{r}h\left(i\right)S_{W_{i}} \end{array}$$ (23)

where *h*(*i*) is a salient degree value histogram, *k* is a constant. Once the value of salient degree *S*_*W*_*i*__ is greater than *Th*, the corresponding region is regarded as a region of interest (ROI). Finally, morphological operation is used to obtain the BM of the interest objects, *BM*_1_ = {*R*_1,1_, ⋯, *R*_1,*q*_1__}, where *q*_1_ is number of the ROIs.

Because motion of interest objects is often nonrigid, each region in *BM*_1_ may not comprise complete structure shapes of the interest objects. To settle such deficiencies, we reuse conspicuity spatial intensity map to get more completed BM. The same operations are performed for conspicuity spatial intensity map (*S*_1_ = 𝓝(*F*_*o*_) + 𝓝(*F*)) to obtain BM including structure shapes of the objects, *BM*_2_ = {*R*_2,1_, ⋯, *R*_2,*q*_2__}. Then, BM of moving objects, *BM*_3_ = {*R*_3,1_, ⋅, *R*_3,*q*_3__}, is achieved by the interaction between both *BM*_1_ and *BM*_2_ as follows:

$$\begin{array}{c} R_{3,c}=\{\begin{array}{cc} R_{1,i}\cup R_{2,j} & \text{if}\;R_{1,i}\cap R_{2,j}\neq \Phi \\ \Phi & \text{others} \end{array} \end{array}$$ (24)

To further refine BM of moving objects, conspicuity motion intensity map (*S*_2_ = 𝓝(*M*_*o*_) + 𝓝(*M*)) is reused and performed with the same operations to reduce regions of still objects. Assume BM from conspicuity motion intensity map as *BM*_4_ = {*R*_4,1_, ⋅, *R*_4,*q*_4__}. Final BM of moving objects, *BM* = {*R*_1_, ⋯, *R*_*q*_} is obtained by the interaction between *BM*_3_ and *BM*_4_ as follows:

$$\begin{array}{c} R_{c}=\{\begin{array}{cc} R_{3,i} & \text{if}\;R_{3,i}\cap R_{4,j}\neq \Phi \\ \Phi & \text{others} \end{array} \end{array}$$ (25)

**Fig 6:**
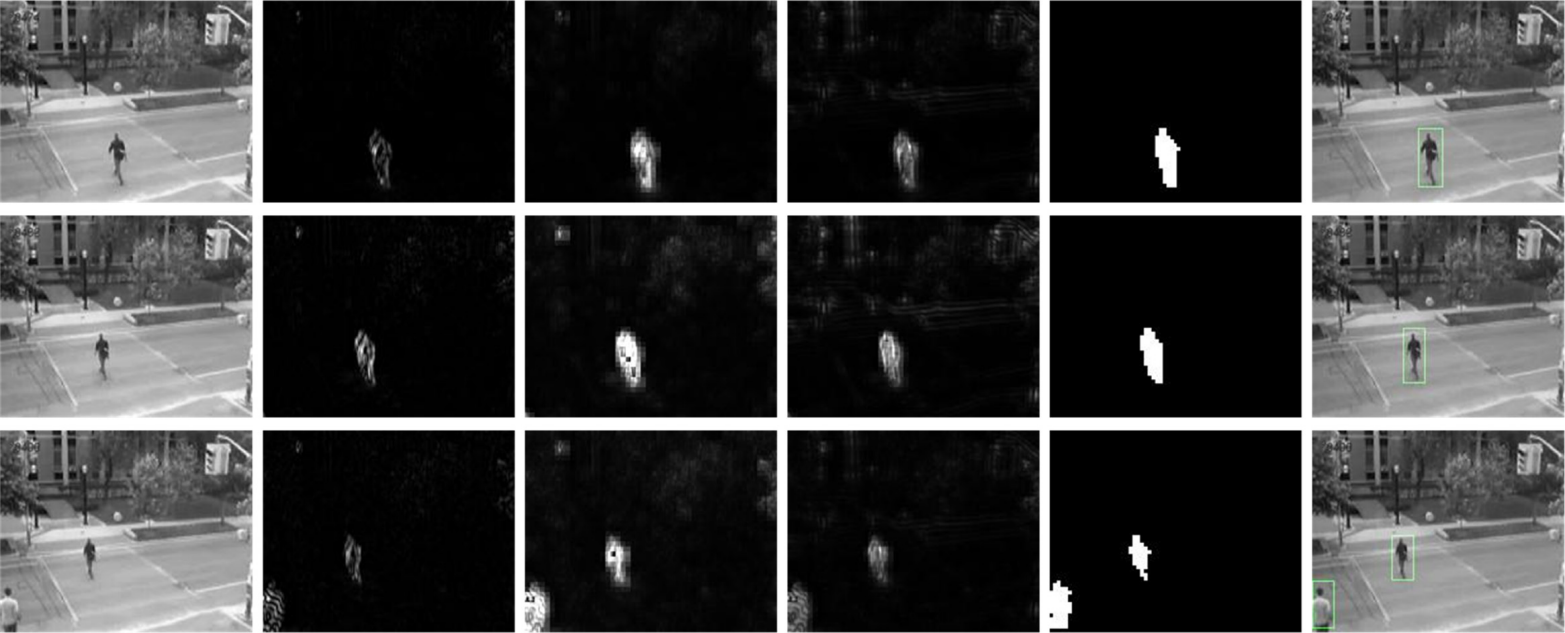
Example of operation of the attention model with a video subsequence. From the first to final column: snapshots of origin sequences, surround suppression energy (with *v* = 0.5*ppF* and *θ* = 0°), perceptual grouping feature maps (with *v* = 0.5*ppF* and *θ* = 0°), saliency maps and binary masks of moving objects, and ground truth rectangles after localization of action objects.

**Fig 7:**
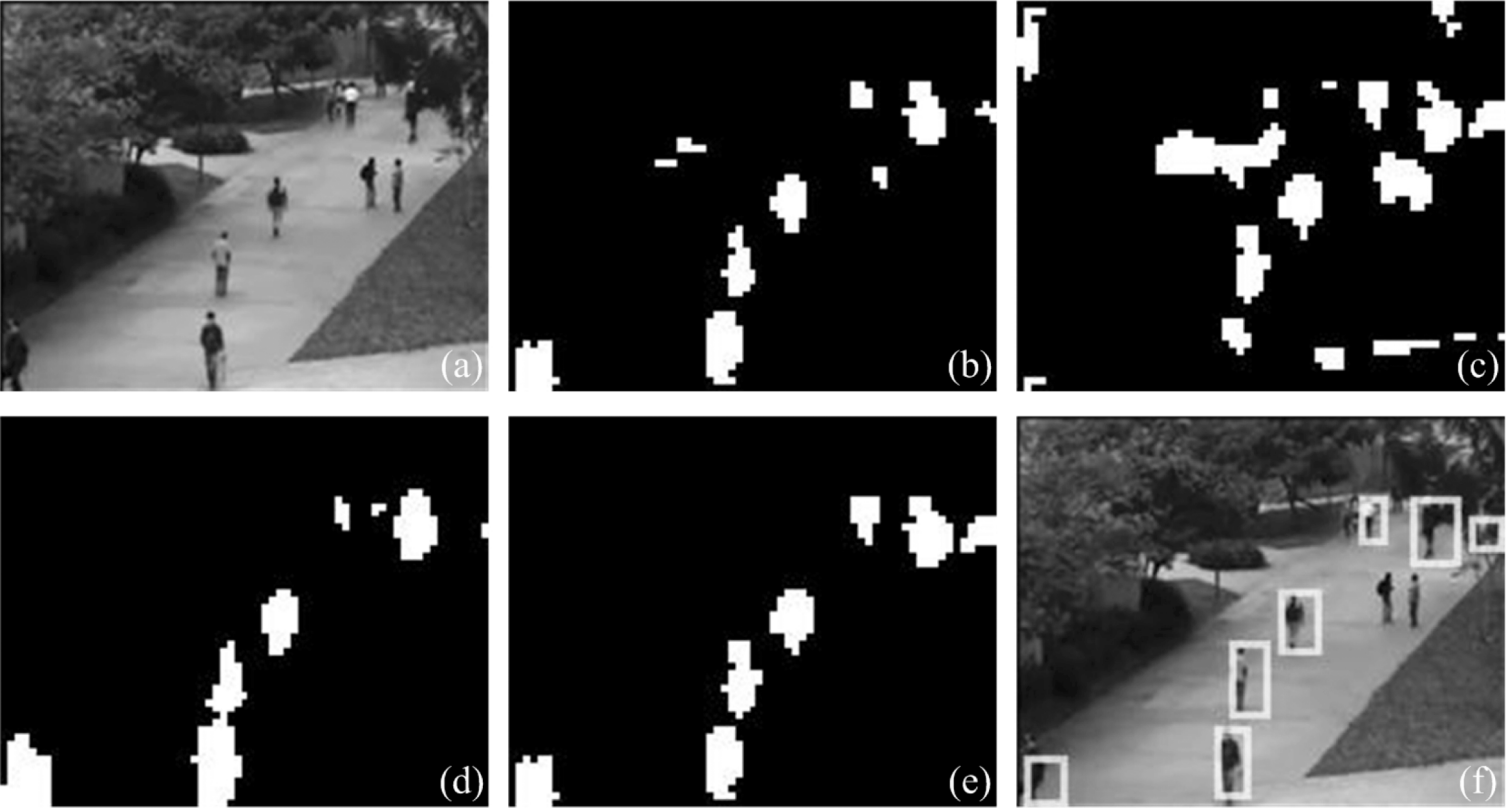
Example of motion object extraction. (a) Snapshot of origin image, (b) BM from saliency map, (c) BM from conspicuity spatial intensity map, (d) BM from conspicuity motion intensity map, (e) BM combining with conspicuity spatial and motion intensity map, (f) ground truth of action objects. Reprinted from [http://www.svcl.ucsd.edu/projects/anomaly/dataset.htm] under a CC BY license, with permission from [Weixin Li], original copyright [2007]. (S1 File).

It can be seen in Fig 6 an example of moving objects detection based on our proposed visual attention model. Fig 7 shows different results detected from the sequences with our attention model in different conditions. Although moving objects can be directly detected from saliency map into BM as shown in Fig 7(b), the parts of still objects, which are high contrast, are also obtained, and only parts of some moving objects are included in BM. If the spatial and motion intensity conspicuity maps are reused in our model, complete structure of moving objects can be achieved and regions of still objects are removed as shown in Fig 7(e).

## Spiking Neuron Network and Action Recognition

In the visual system, perceptual information also requires serial processing for visual tasks [37]. The rest of the model proposed is arranged into two main phases: (1) Spiking layer, which transforms spatiotemporal information detected into spikes train through spiking neuron model; (2) Motion analysis, where spiking train is analyzed to extract features which can represent action behavior.

### 1 Neuron Distribution

**Fig 8:**
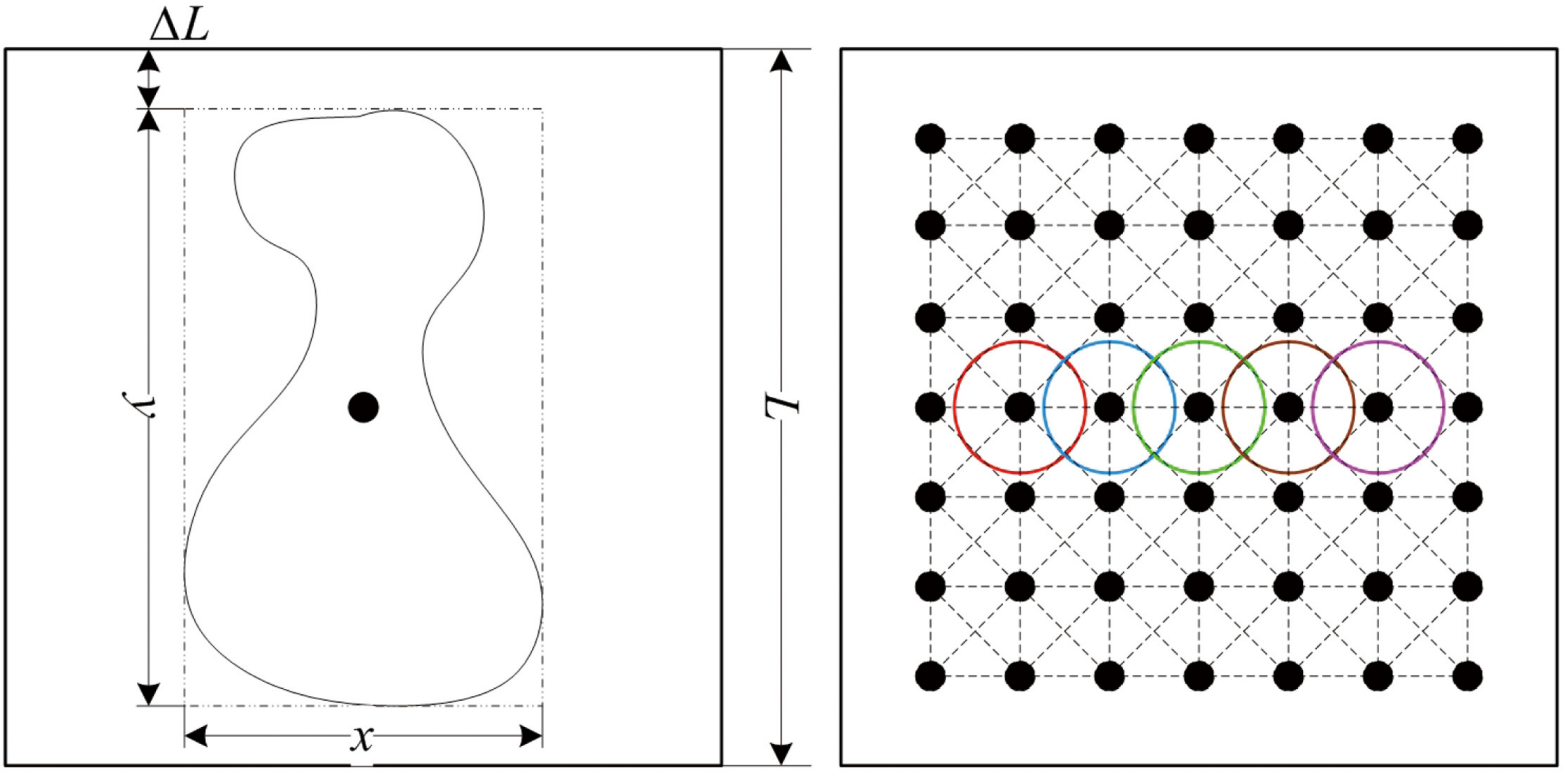
Distribution schematics for spiking neuron. *L* is maximum size of attentional visual field, in which neurons connect to each other to form a network. A block point is a center position of a receptive field, range of which is represented by a color circle.

Visual attention enables a salient object to be processed within the limited area of the visual field, called as “field of attention” (FA) [52]. Therefore, the salient object as motion stimulus is firstly mapped into the central region of the retina, called as fovea, then mapped into visual cortex by several steps along the visual pathway. Though the distribution of receptor cells on the retina is like a Gaussian function with a small variance around the optical axis [53], the fovea has the highest acuity and cell density. To this end, we assume that the distribution of receptor cells in the fovea is uniform. Accordingly, the distribution of the V1 cells in FA bounded area is also uniform, as shown Fig 8. A black spot in the distribution map represents single spiking neuron and the color circle indicates its CRF.

Due to non-rigid motion and scale change of the salient object in sequence, the size and center of the FA change with its BM. We consider FA area as a square with sides of length *L* and central position **x**_*c*_. The length of *L* is defined as follows:

$$\begin{array}{c} L=\max_{}\left\{l_{x},l_{y}\right\}+\Delta L \end{array}$$ (26)

where *l*_*x*_ and *l*_*y*_ are width and height of the BM bounded area, respectively. Δ*L* is extending spatial extent, which is set *n*_1_ times of a constant *r*, thus ensuring the BM completely embedded in FA, as shown in Fig 8. In generally, due to the continuous movement of the salient object in sequence, *L*(*t*) is a time-varying function. To avoid frequent changes, *L*(*t*) is constrained by follows:

$$\begin{array}{c} L\left(t\right)=L\left(t_{0}\right)+\left\lfloor \left(\max_{t}\left\{l_{x},l_{y}\right\}-\max_{t_{0}}\left\{l_{x},l_{y}\right\}\right)/\left(n_{2}r\right)\right\rfloor \end{array}$$ (27)

where *t* is present time and *t*_0_ is last time when *L*(*t*) is updated. *n*_2_ is a factor constant, constrained by *n*_2_ < *n*_1_.

On the other hand, the visual attention is able to track the salient object in motion and to keep it in the foveal region, known as smooth pursuit [17]. It makes FA center position **x**_*c*_ be almost identical with BM geometer center **x**_*b*_. Similar to above method, **x**_*c*_ can be determined by **x**_*b*_ as follows:

$$\begin{array}{c} x_{c}\left(t\right)=\{\begin{array}{cc} x_{b}\left(t\right) & \text{if}\;\;|x_{b}\left(t\right)-x_{c}\left(t_{0}\right)|\geq n_{3}r\\ x_{c}\left(t_{0}\right) & \text{others} \end{array} \end{array}$$ (28)

where *n*_3_ is another factor constant. The constraint of *n*_2_ + *n*_3_ < *n*_1_ ensures BM within FA bounded area. In this paper, *n*_1_, *n*_2_, *n*_3_ are respectively set as 7, 2 and 2.

Finally, the original video streams are resized and centered to produce sequences of 120 × 120 pixels according to FA bounded areas. The spatiotemporal information falling in the FA is further processed by V1 cells. We consider *N*_*v*_ layers of organized V1 cells, each of which is built with the V1 cells with the same properties of spatial-temporal tuning. The RF of V1 cell at the physical position **x**_*i*_ is defined by its properties of spatial-temporal tuning. Each layer is consist of *N*_*o*_ + 1 sub-layers with *N*_*o*_ different orientations and non-orientation. In the physical position, where RF of cells is centered, one column is formed in each layer, which has as many elements as *N*_*o*_ + 1 orientations defined. Therefore, for all layers, there are *N*_*v*_ × (*N*_*o*_ + 1) cells along *N*_*v*_ layers in **x**_*i*_.

### 2 Spiking Neuron Model

A typical neuron is synaptically linked with hundreds of thousands of others. To capture functional properties and realistic dynamic behaviors, a spiking neuron is always described by computational model according to biological plausibility and the computational efficiency. So, many models have been proposed to simulate the entity in the literature [54].

In this paper, we use conductance-driven integrate and fire neuron model (IF model) [38] to simulate spiking neurons. The formula is as follows:

$$\begin{array}{cc} \frac{du_{i}\left(t\right)}{dt}= & G_{i}^{E}\left(t\right)\left(V^{E}-u_{i}\left(t\right)\right)+G_{i}^{I}\left(t\right)\left(V^{I}-u_{i}\left(t\right)\right)\\ & +g^{L}\left(V^{L}-u_{i}\left(t\right)\right)+I_{i}\left(t\right) \end{array}$$ (29)

where $G_{i}^{E}(t)$ is the normalized excitatory conductance directly associated with the pre-synaptic neurons connected neuron *i*, and $G_{i}^{I}(t)$ is an inhibitory normalized conductance; The conductance *g* ^*L*^ is the passive leaks in the cell’s membrane; *I*_*i*_(*t*) is an external input current. When the normalized membrane potential *u*_*i*_(*t*) ≥ *u*_0_, spiking neuron *i* will emit a spike and the voltage reset to the resting potential. As some properties of the cells in V1 are used to detect spatiotemporal information, the first and second terms corresponding to $G_{i}^{I}(t)$ and $G_{i}^{E}(t)$ in Eq (29) as internal current are integrated into *I*_*i*_(*t*) here. Eq (29) is rewritten as

$$\begin{array}{c} \frac{du_{i}\left(t\right)}{dt}=g^{L}\left(V^{L}-u_{i}\left(t\right)\right)+I_{i}\left(t\right) \end{array}$$ (30)

The typical values for *V* ^*L*^ is -70*mv*.

### 3 Neuron’s Input

Objective of the spiking neuron model described above is to transform the analogous response of V1 cell defined in Eq (12) to the spiking response so as to characterize the activity of a neuron. From Eq (30), the activity of a neuron is determined by external input current *I*_*i*_(*t*) of the the spiking neuron and the membrane potential threshold.

First, let us consider input of a spiking neuron *i* in V1 whose center is located in **x**_*i*_. Its external input current *I*_*i*_(*t*) associates with the analogous response of V1 cell defined in Eq (12). However, the activation of the cell is in range of classical RF. The computational operator over RF in a sub-layer (e.g. same preferred motion direction and speed) is needed [55]. Thus, the *input current* *I*_*i*_(*t*) of the *i*th neuron is modeled in Eq (31) as follows:

$$\begin{array}{c} I_{i}\left(t\right)=K_{exc}\;\max_{i}\left\{R_{v,(\theta )}\left(x,t\right)\right\} \end{array}$$ (31)

where *K*_*exc*_ is an amplification factor, *R*_*v*,(*θ*)_(**x**, *t*) refers to V1 cell response defined in Eq (12) with *k* = 4 and max_*i*_ is a operator of local maximum [56].

### 4 Spike Train Analysis for Action Recognition

According to above description, every spiking neuron in V1 generates a series of spikes corresponding to stimuli of human action over time, called spike train *η*_*i*_(*t*). To recognize human action, we only need to analyze the activity of spiking networks built by spiking neurons in V1 cortex, so that features representing human action can be extracted from spike trains. For a spike train, it comprises of discrete events in time, can be described by succession of emission times of a spiking neuron in V1 as $\eta_{i}(t)=\{\cdots ,t_{i}^{n},\cdots \}$, where $t_{i}^{n}$ corresponds to the *n*th spike of the neuron of index *i*.

Since our main purpose focuses on action recognition based on the proposed framework rather than strategies of spike-based code, some methods about high-level statistics of spike trains [57] are not considered in this paper. Similar to [13], mean firing rate over time, which is one of the most general and effective methods, is used.

**Fig 9:**
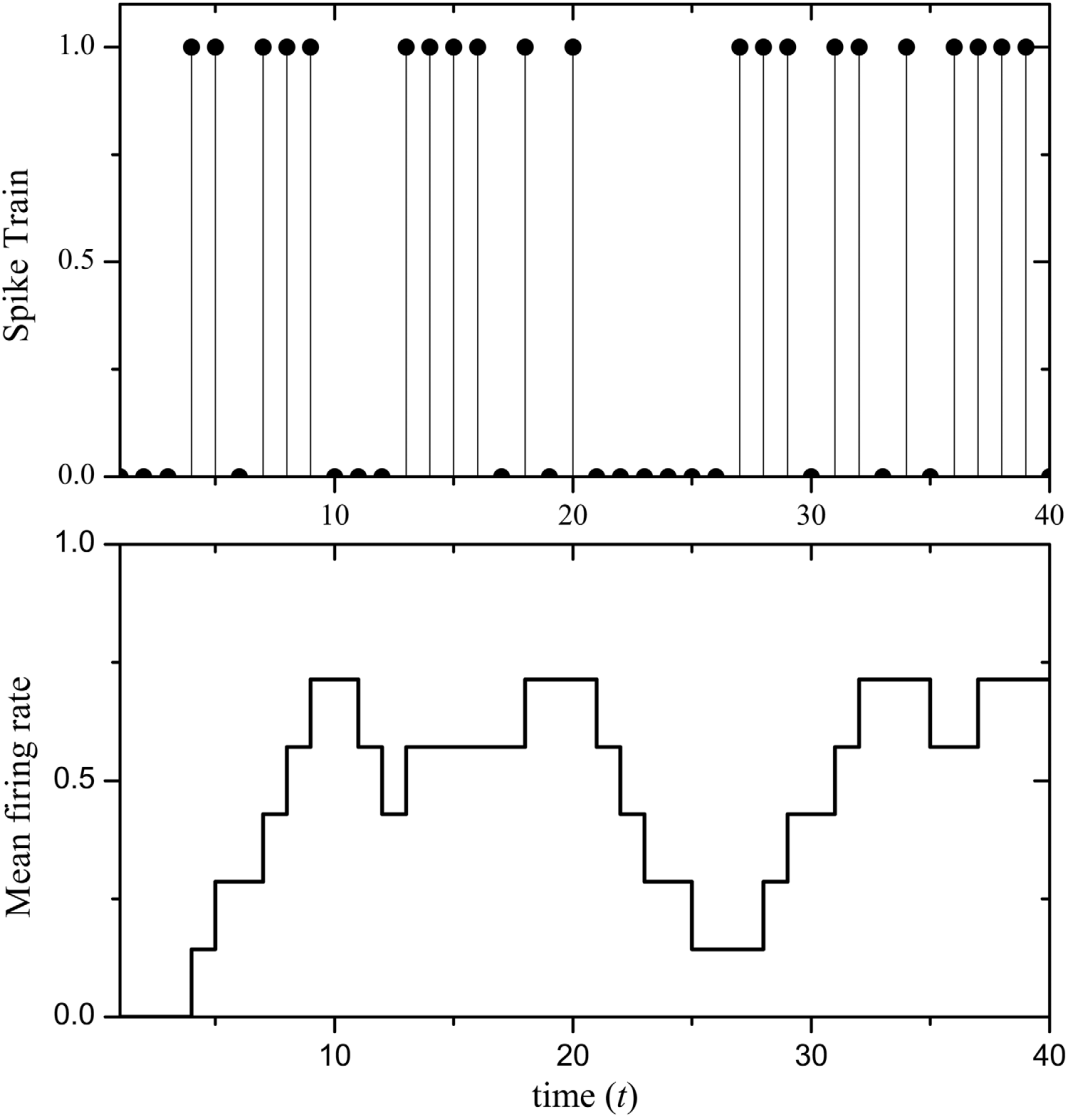
Spike train (*upper*) and its Mean firing rate (*bottom*).

**Fig 10:**
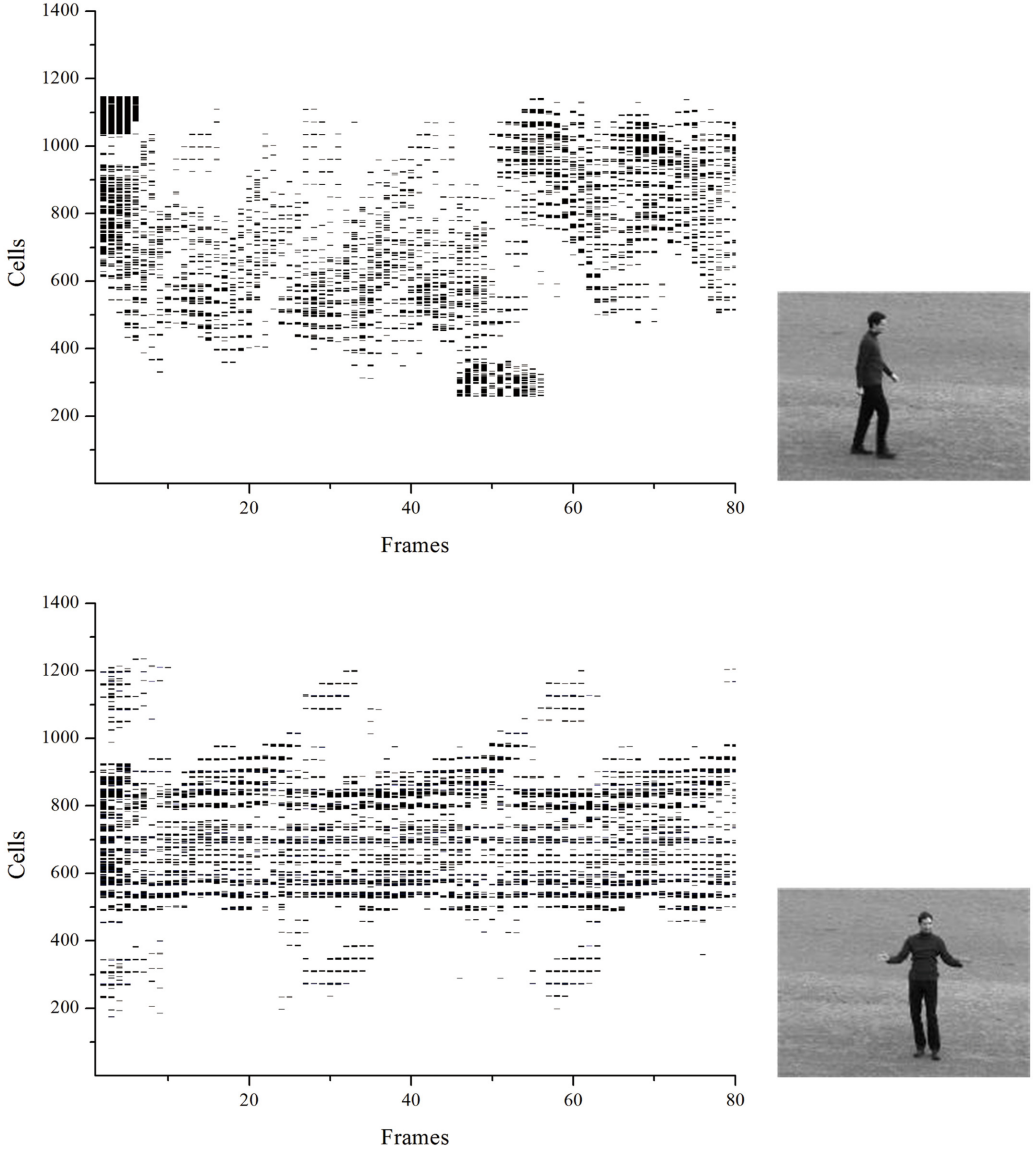
Raster plots obtained considering the 1400 spiking neuron cells in two different actions shown at *right*: walking and handclapping under condition 1 in KTH.

For a spiking neuron, its mean firing rate over time is computed with the average number of spikes inside a temporal window, Eq (32) defined as:

$$\begin{array}{c} {𝓣}_{i}\left(t,\Delta t\right)=\frac{\eta_{i}\left(t-\Delta t,t\right)}{\Delta t} \end{array}$$ (32)

where *η*_*i*_(*t* − Δ*t*, *t*) counts the number of spikes emitted by neuron *i* inside the glide time window Δ*t*. Fig 9 displays the spike train of a neuron and its mean firing rate map, where Δ*t* = 7. Fig 10 shows raster plots obtained considering the 1400 cells of a given orientation in two different actions: walking and handclapping.

In Eq (32) and Fig 9, the estimation of the mean firing rate depends on the size of the glide time window. A wider window Δ*t* can reduce the individual spike generated by noise stimuli resulting in smooth curve of mean firing rate, but it simultaneously degrates the significance in time. Although the smaller can highlight instantaneous firing rate, it also emphasizes the uncertainty of the spike train corresponding to dynamic stimulus. To do this, we will select a suitable size of the glide time window to measure the mean firing rate according to our given vision application.

Another problem for rate coding stems from the fact that the firing rate distribution of real neurons is not flat, but rather heavily skews towards low firing rates. In order to effectively express activity of a spiking neuron *i* corresponding to the stimuli of human action as the process of human acting or doing, a cumulative mean firing rate 𝓣_*i*_(*t*, Δ*t*) is defined as follows:

$$\begin{array}{c} {𝓣}_{i}=\frac{\sum_{t=1}^{t_{max}}{𝓣}_{i}\left(t,\Delta t\right)}{t_{max}} \end{array}$$ (33)

where *t*_*max*_ is length of the subsequences encoded.

Remarkably, it will be of limited use at the very least for the cumulative mean firing rates of individual neuron to code action pattern. To represent the human action, the activities of all spiking neurons in FA should be regarded as an entity, rather than considering each neuron independently. Correspondingly, we define the mean motion map 𝓜_*v*,(*θ*)_ at preferred speed and orientation corresponding to the input stimulus *I*(**x**, *t*) by

$$\begin{array}{c} {𝓜}_{v,(\theta )}=\left\{{𝓣}_{p}\right\};\;p=1,\cdots ,N_{c} \end{array}$$ (34)

where *N*_*c*_ is the number of V1 cells per sub-layer. Because the mean motion map includes the mean activities of all spiking neuron in FA excited by stimuli from human action, and it represents action process, we call it as *action encode*.

Due to *N*_*o*_ + 1 orientation (including non-orientation) in each layer, *N*_*o*_ + 1 mean motion maps is built. So, we use all mean motion maps as feature vectors to encode human action. The feature vectors can be defined as:

$$\begin{array}{c} H_{I}=\left\{{𝓜}_{j}\right\};\;j=1,\cdots ,N_{v}\times \left(N_{o}+1\right) \end{array}$$ (35)

where *N*_*v*_ is the number of different speed layers, Then using V1 model, feature vector *H*_*I*_ extracted from video sequence *I*(**x**, *t*) is input into classifier for action recognition.

Classifying is the final step in action recognition. Classifier as the mathematical model is used to classify the actions. The selection of classifier is directly related to the recognition results. In this paper, we use supervised learning method, i.e. support vector machine (SVM), to recognize actions in data sets.

## Materials and Methods

### 1 Database

In our experiments, three publicly available datasets are tested, which are Weizmann (http://www.wisdom.weizmann.ac.il/vision/SpaceTimeActions.html), KTH (http://www.nada.kth.se/cvap/actions) and UCF Sports (http://vision.eecs.ucf.edu/data.html). Weizmann human action data set includes 81 video sequences with 9 types of single person actions performed by nine subjects: running (run), walking (walk), jumping-jack (jack), jumping forward on two legs (jump), jumping in place on two legs (pjump), galloping-sideways (side), waving two hands (wave2), waving one hand (wave1), and bending (bend).

KTH data set consists of 150 video sequences with 25 subjects performing six types of single person actions: walking, jogging, running, boxing, hand waving (handwave) and hand clapping (handclap). These actions are performed several times by twenty-five subjects in four different conditions: outdoors (s1), outdoors with scale variation (s2), outdoors with different clothes (s3) and indoors with lighting variation (s4). The sequences are down-sampled to a spatial resolution of 160 × 120 pixels.

UCF Sports data set includes diving, golf swinging, kicking, lifting, horseback riding, running, skating, swinging a baseball bat, and pole vaulting. The dataset contains over 200 video sequences at a resolution of 720 × 480 pixels. The collection represents a natural pool of actions featured in a wide range of scenes and view points.

### 2 Parameter setting

Our proposed model is constructed with *N*_*v*_ layers of preferred speeds and each layer is composed of five sub-layers corresponding to five orientations (0°, 45°, 90°, 135°, and a non-orientation). As the preferred speeds at which the model runs are associated with spatial-temporal frequency and computing load, their number and values will be determined by experimental results. The parameter settings can be seen in Table 1. The model has a total of 5*N*_*v*_ sub-layers, formed by 5 orientations (including a non-orientation) and *N*_*v*_ different spatial-temporal tunings. There is a total of 1600 cells in a sub-layer, being distributed in the whole FA. It is noted that the FAs generated by our attention model are resized and centered in 120 × 120 pixels, forming new FA sequences. The sizes of receptive field patch and surrounding area are 2*σ* and 8*σ* respectively.

**Table 1:** Parameters Used for V1 Mode.

Parameters	Values
FA size	120 pixels
Number of preferred speeds	*N*_*v*_
Number of preferred orientations	5
Neuron density	0.33 per pixel
Size of receptive field patch	2*σ* pixels
Size of surrounding area	8*σ* pixels
Number of neurons per sub-layer	1600

To compare the performance with other methods, we conduct experiments on all of the three given datasets under the following three experimental setups:

- Setup 1 is that one sequence of a subject is selected as the testing data while the sequences of other subjects are employed as the training data, called leave-one-out cross validation similar to [31].
- Setup 2 uses the sequences of more than one subjects for testing and others for training [13] and [5]. We select 6 random subjects as a training set and the remaining 3 subjects as a testing set for Weizmann dataset, and 16 subjects randomly drawn from KTH dataset for training and the remaining 9 subjects for testing. We run all the possible training sets (84) for Weizmann and do 100 trails for KTH
- Setup 3 is similar to setup 2, but only do five random trails, following the same experimental protocol described in Jhuang et al. [4].

Each setup examines the ability of the proposed approach to recognize human actions in videos. The performance is based on the average of all trails. It is noted that this is done separately for each scene (s1, s2, s3, or s4) in KTH dataset.

## Experimental Results

Extensive experiments have been carried out to verify the effectiveness of the proposed approach. The following describes the details of the experiments and the results.

### 1 Effects of Different Parameter Sets on the Performance

In our model, the feature vector *H*_*I*_ computed in Eq (35), is dependent on different parameters, including subsequence length *t*_*max*_, size of glide time window △*t*, number of preferred speeds *N*_*v*_ and their values, et al. To evaluate the performance of our model for action recognition, the following test experiments are firstly performed with different parameter settings. Moreover, all experiments are implemented under Setup 1 in order to ensure the consistency and comparability.

#### Frame length

**Fig 11:**
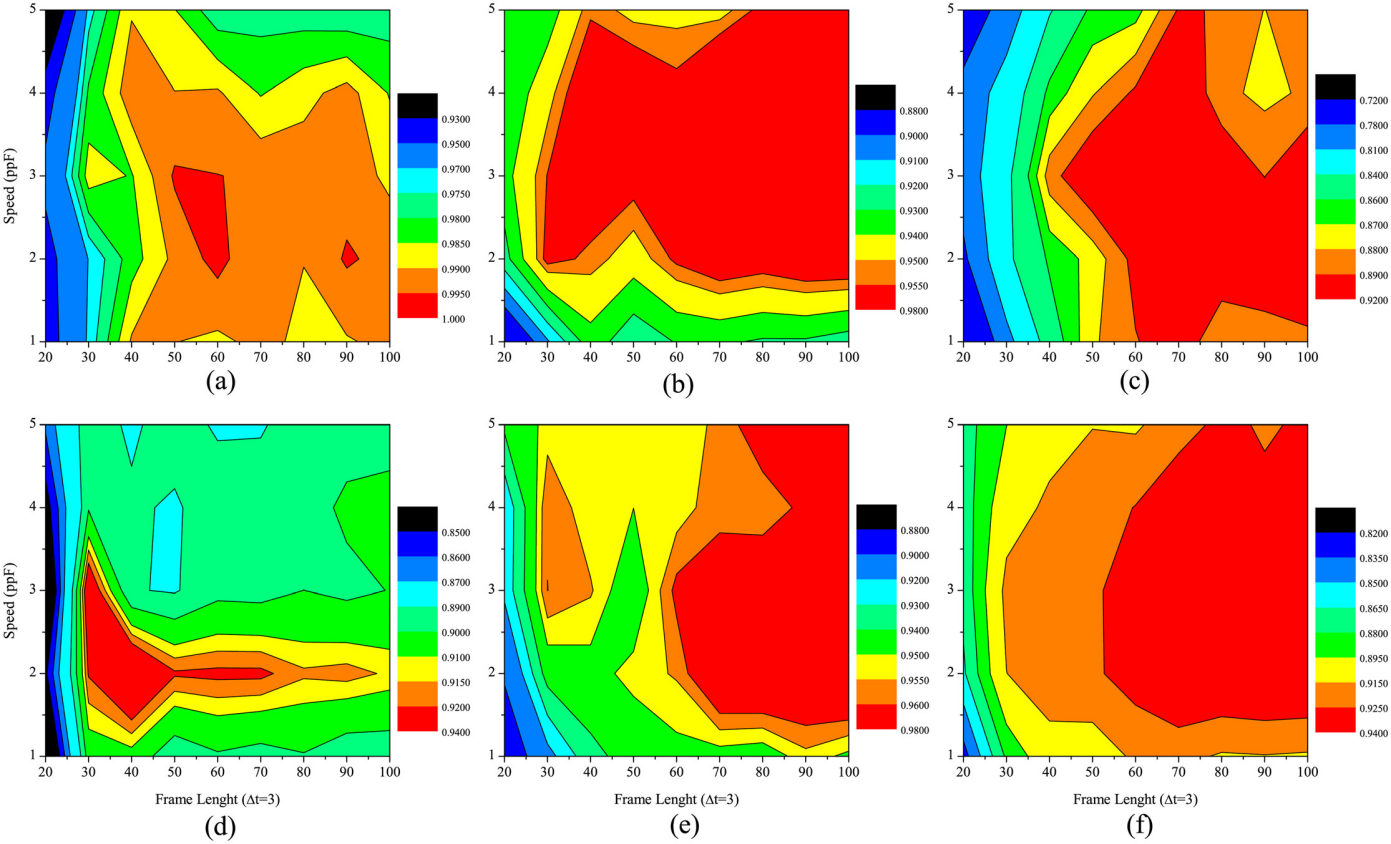
The average recognition rates proposed model with different frame lengths and different speeds for different datasets, which size of glide time window is set as a constant value of 3. (a)Weizimann, (b)KTH(s1),(c) KTH(s2), (d) KTH(s3), (e) KTH(s4) and (f) average of KTH (all conditions).

Firstly, to examine the impact of the frame length of the selected subsequence *t*_*max*_ on the recognition results, we apply the classifier SVM to assess the proposed model on all subsequences randomly selected from all original videos of Weizmann and KTH datasets. Note that all tests are performed at five different speeds *v*, such as 1, 2, 3, 4 and 5 *ppF*, with the size of glide time window △*t* = 3. The classifying results with different parameter sets are shown in Fig 11, which indicates that: (1) the average recognition rates (ARRs) increase with increment of subsequence length *t*_*max*_ from 20 to 100; (2) ARR on each of test datasets is different at different preferred speeds; (3) ARRs on different test datasets are different at each of the preferred speeds.

**Fig 12:**
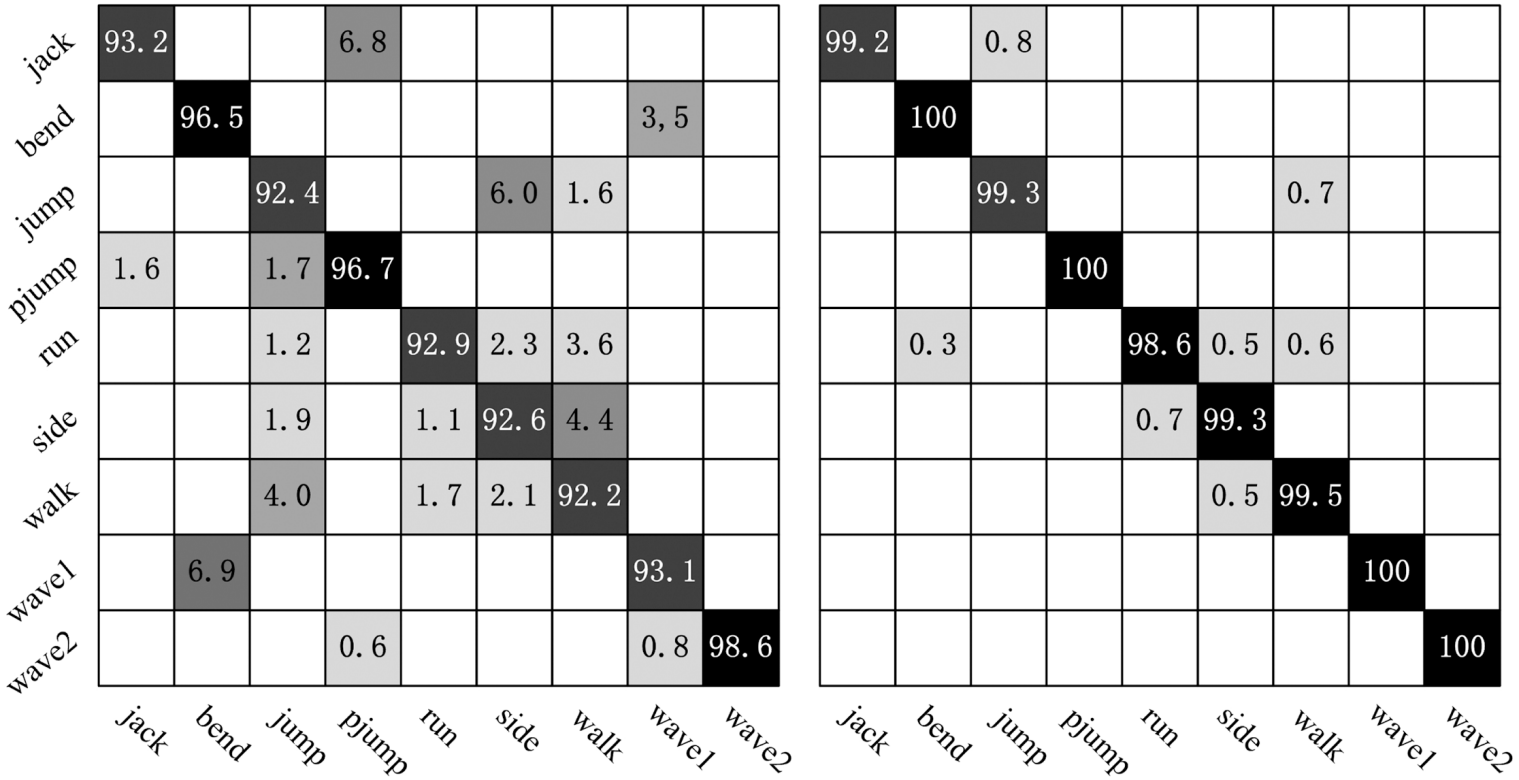
Confusion matrices obtained using two different frame lengths at preferred speed *v* = 2*ppF*: *Left* 20 frames, and *Right* 60 frames on Weizmann dataset.

How long subsequence is suitable for action recognition? We analyze the test results on Weizmann dataset. From Fig 11, it can be clearly seen that the ARR rapidly increases with the frame length of selected subsequence at the beginning. For example, the ARR on Weizmann dataset is only 94.26% with the frame length of 20 at preferred speed *v* = 2*ppF*, whereas the ARR rapidly raises to 98.27% at the frame length of 40, then keeps relatively stable at the length more than 40. In order to obtain a better understanding of this phenomenon, we estimate the confusion matrices for the 81 sequences from Weizmann dataset (See in Fig 12). From a qualitative comparison between the performance of the human action recognition at the frame length of 20 and 60, we find that ARRs for actions are related to their characteristics, such as average cycle (frame length of a whole action), deviation (see Table 2). The ARRs of all actions are improved significantly when the frame length is 60, as illustrated in Fig 12. The reason mainly is that the length of average cycles for all actions is not more than 60 frames. Certainly, it can be observed that the larger the frame length is, the more information is encoded, which is helpful for action recognition. Moreover, it is relatively significant that the performance can be improved for actions with small relative deviations to average cycles.

**Table 2:** Average Cycles of Actions in Weizmann and KTH Dataset.

Weizmann			KHT		
Class	Cycle	Num.(≥ 40)	Class	Cycle	Num.(≥ 40)
runn	20.3 ±3	0	walking	27.7 ± 4	0
walk	26.9 ±2	0	jogging	29.9 ± 4	0
jack	27.2 ±3	0	running	17.0 ± 4	0
jump	13.4 ±3	0	boxing	31.7 ± 20	5
pjump	16.1 ±3	0	handwave	41.5 ± 28	1
side	15.0 ±2	0	handclap	27.8 ± 16	12
wave2	29.2 ±4	0			
wave1	29.0 ±4	0			
bending	60.9 ±7	9			
average	25.0			27.6	

The same test on KTH dataset is performed and the experimental results under four different conditions are shown in Fig 11(b)–11(e). The same conclusion can be obtained: ARRs increase with increment of the frame length and keep relatively stable at the length more than 60 frames. It is obvious for overall ARRs under all conditions at different speeds shown in Fig 11(f). Considering the computational load increasing with the growing frame length, as a compromise plan, maximum frame length of the subsequence selected from original videos is set to 60 frames for all following experiments.

#### Size of glide time window

Secondly, to evaluate the influence of the size of glide time window Δ*t* in Eq (33) on the recognition results, we perform the same test on Weizmann and KTH datasets (s2, s3 and s4). It is noted that the maximum frame length is 60 for all subsequences randomly selected from original videos for training and testing and the SVM based on Gaussian kernel is used as a classifier which discriminates action classes from others.

**Fig 13:**
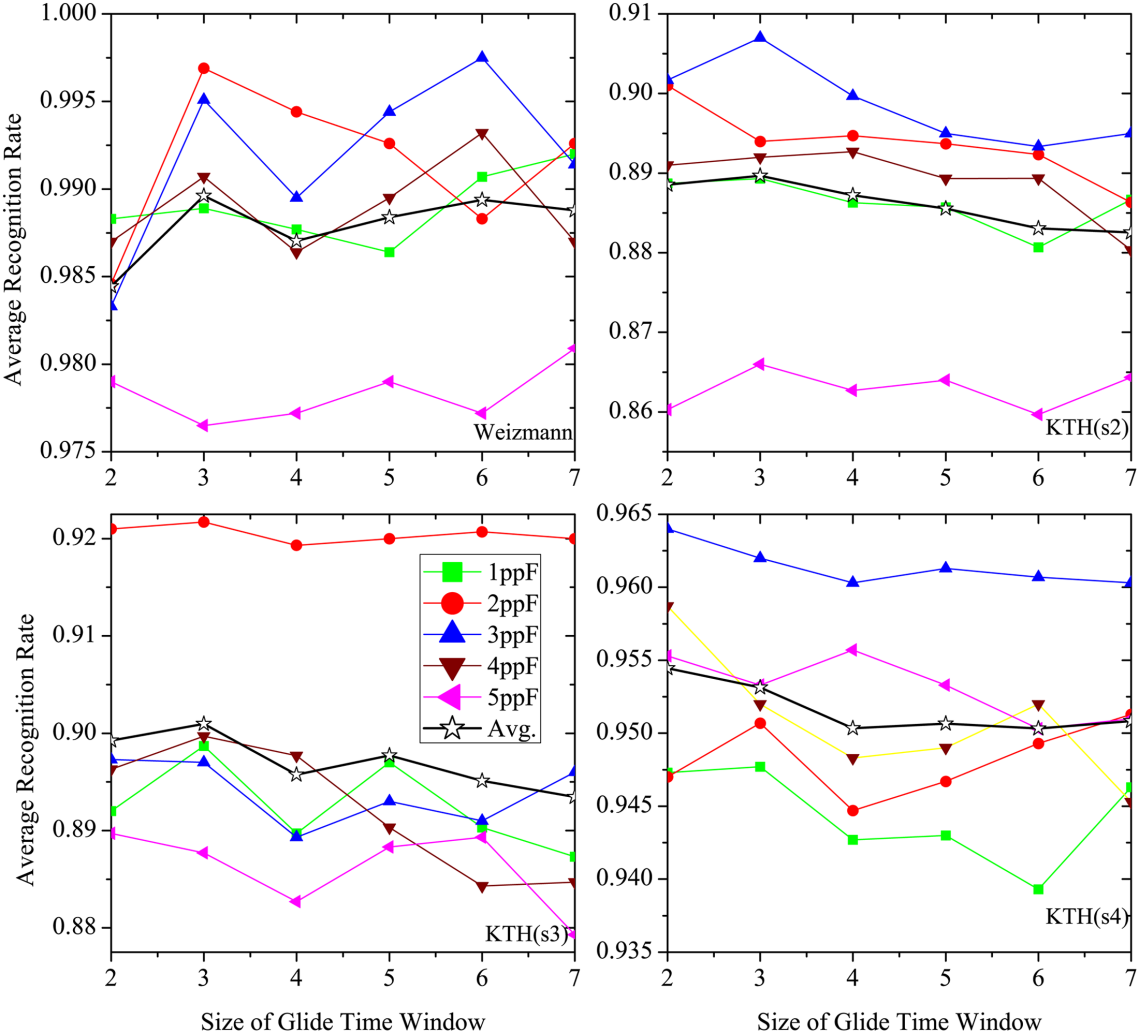
The average recognition rate of proposed model with different sizes of glide time window and different speeds for various datasets, where maximum frame length is set as constant value of 60. From *upper left* to *lower right*, the sub-figures correspond to the cases of Weizimann, KTH(s2), KTH(s3), KTH(s4), respectively.

Fig 13 shows experimental results with different size values of glide time window at different preferred speeds. It is seen that the ARRs at different speeds on each dataset (including each condition) vary with size of glide time window. Considering performance at all speeds used in test, we find that the optimal window size value is 3 in most cases. It also indicates that the features computed with different sizes of glide time window also affect the recognition performance. The mean motion maps are easily interrupted by undesired stimulus when the window size is small, whereas the distinctiveness of feature vectors among human actions are degraded in large window size. According to the average ARRs at all speeds from the experimental results shown in Fig 13, the size of glide time window is set to 3.

#### Number of the preferred speeds and their values

The experimental results shown in Figs 11 and 13 exhibit distinct recognition performance at different speeds. For example, the highest ARR on KTH dataset (s2) is provided at the preferred speed of *v* = 3*ppF* (Δ*t* = 3), whereas the actions on KTH dataset (s3) are more accurately classified at the preferred speed of *v* = 2*ppF*. As the different human actions operate at the different speeds and the same action in different scales also does with different speeds, number of the preferred speeds and their values employed to compute action features will greatly affect the recognition results.

**Fig 14:**
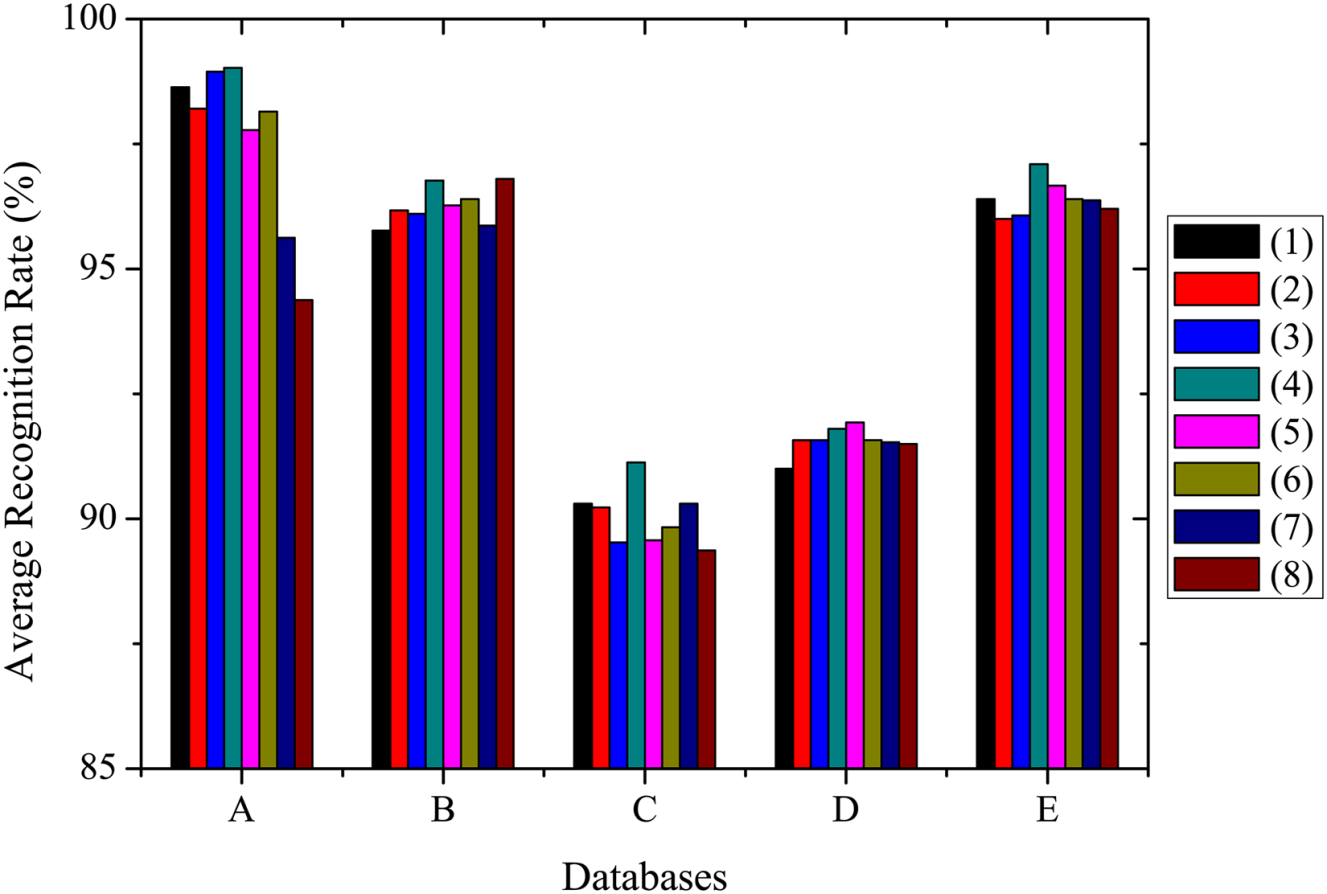
The average recognition rates of the proposed model at combination of different speeds. A. Weizmann, B. KTH(s1), C. KTH(s2), D. KTH(s3), and E. KTH(s4). The labels from 1 to 8 represent the speed combinations of 2+3, 2+3+4, 1+2+3, 1+3, 1+2+3+4+5, 2+3+4+5, 1+2+4, and 1+2+5, respectively.

However, it is impossible to detect features at all different speeds to evaluate the influence of preferred speeds on human action recognition due to huge computational cost. Moreover, only choosing one preferred speed for action recognition is not reasonable because of the complexity of action. To obtain more accurate recognition performance, we need to evaluate how many and which preferred speeds should be introduced into our model to extract motion features for human action recognition in general videos. It is known that most real-world video sequences have a center-biased motion vector distribution. More than 70 to 80% of the motion vectors can be regarded as quasi-stationary and most of the motion vectors are enclosed in the central 5 × 5 area [58]. Therefore, we opt to evaluate the performance of our model with combination of different speeds of which the value is no more than 5. For simple computation, the speed is set to integer value. Because the combinations of different speeds are too more, the experimental results on Weizmann and KTH datasets at some combinations are shown in Fig 14. It is clearly seen that the different combinations in our model have an important effect on the accuracy of action recognition. For example, the recognition performance at the combination of two speeds 1+3*ppF* is the best one datasets except KTH (s3) dataset, and is better than that at most combinations on KTH (s3) dataset. The average recognition rate at this combination on all datasets achieves 95.16% and is the best. In view of computation and consideration for overall performance of our model on all datasets, action recognition is performed with the combination of two speeds (1 and 3*ppF*) for all experiments.

### 2 Effects of Different Visual Processing Procedure on the Performance

In order to investigate the behavior of our model with real-world stimuli under two conditions: (1) surround inhibition and (2) field of attention and center localization of human action, all experiments are still performed on Weizmann and KTH datasets with a combination of 2 levels of V1 neurons (*N*_*v*_ = 2, *v* = 1, 3*ppF*), 4 different orientations per level, Δ*t* = 3 and *t*_*max*_ = 60. To evaluate robustness of our model, input sequences with perturbations are used for test under same parameter set. Training and testing sets are arranged with Setup 1.

#### 3D Gabor

3D Gabor filers modeling the spatiotemporal properties of V1 simple cells are crucial to detection of spatiotemporal information from image sequences, which directly affect subsequent extraction of the spatiotemporal features. To examine the advantage of inseparable properties of V1 cells in space and time for human action recognition, we compare the results of our model to those of our model using traditional 2D Gabor filters to replace 3D Gabor filters. In all experiments, to keep the fairness, the spatial scales of 2D Gabor filters are the results computed by Eq (2), other parameters in the model remain the same. The experimental results are listed in Table 3. Results show that our model significantly outperforms the model with traditional 2D Gabor, especially on datasets including complex scenes, such as KTH s2 and s3.

**Table 3:** Performance Comparison with the Model Using 2D Gabor.

Dataset	Weizmann	KTH(s1)	KTH(s2)	KTH(s3)	KTH(s4)	Avg.
3D Gabor	99.02	96.77	91.13	91.80	97.10	95.16
2D Gabor	96.31	93.06	85.18	84.42	93.22	90.44

#### Surround inhibition

**Fig 15:**
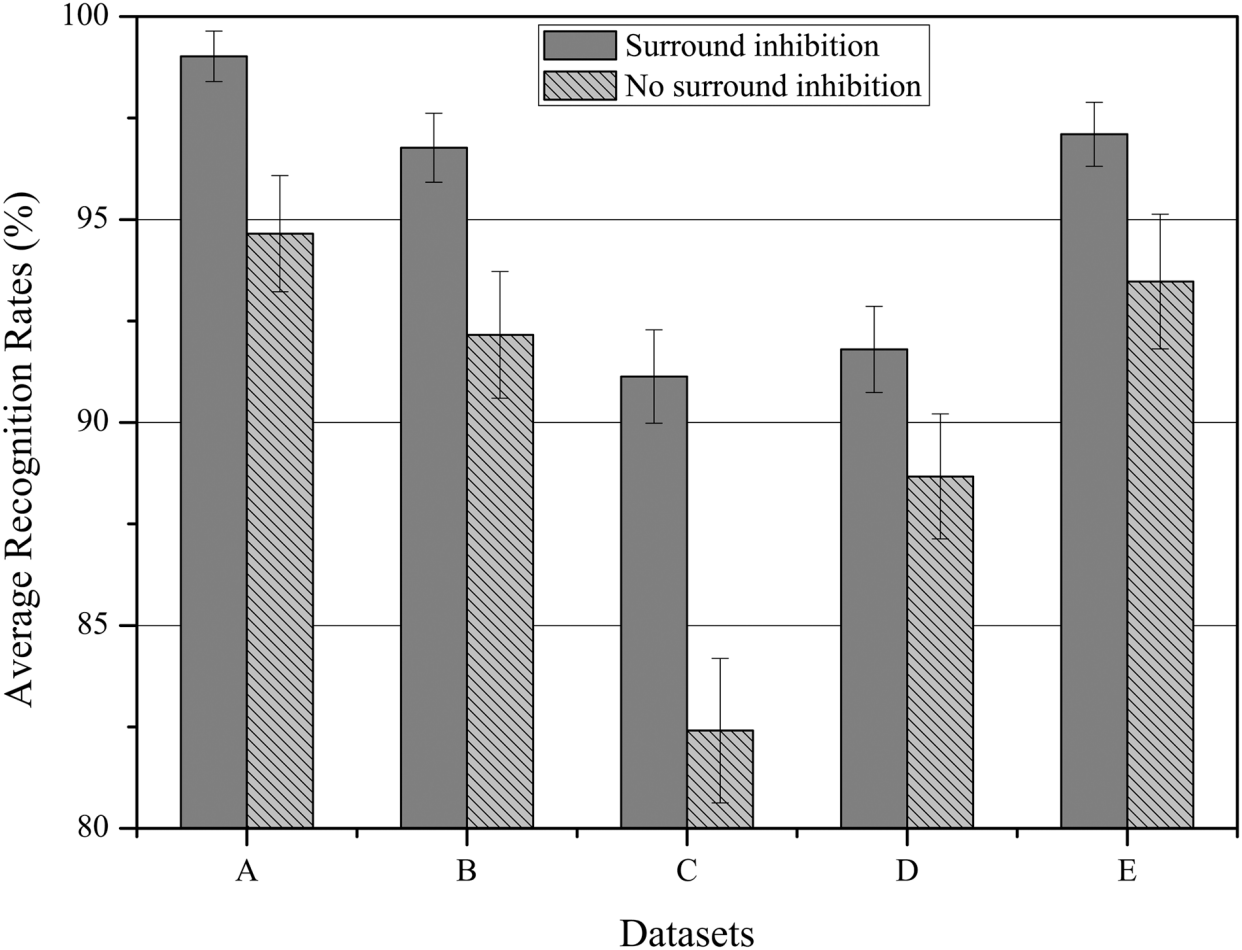
Histograms representing the average recognition rates obtained by our model with 2 conditions: (1) surround inhibition and (2) no surround inhibition on Weizmann and KTH datasets. *A*. Weizmann, *B*. KTH(s1), *C*. KTH(s2), *D*. KTH(s3), *E*. KTH(s4)

To validate the effects of the surround inhibition on our model, we use ${\hat{r}}_{v,(\theta )}(\text{x},t)$ in Eqs (7) and (8) as input of integrate-fire model in Eq (29) to replace *R*_*v*,(*θ*)_(**x**, *t*) in Eq (31). For each training and testing sets, the experiment is performed two times: only considering the activation of the classical RF, and the activation of RF with the surround inhibition proposed. We construct a histogram with the different ARRs obtained by our approach in two cases (Fig 15). As we can see in Fig 15, the values of ARR with the surround inhibition are much higher than no surround inhibition on Weizmann and KTH datasets. At the same time, ARR values with no surround inhibition have a strong variability and the recognition performance highly depends on the sequences used to construct the training set, while the values with surround inhibition are relatively stable.

#### Field of attention and center localization

The attention computational model described in the preceding section is introduced in our action recognition model. The binary masking (BM) of an action object is obtained to determine the center position and size of FA based on our attention model. There are many methods to evaluate the performance of the attention model in terms of correct detections, detection failures, matching area, and so on. In our case, the aim is not to emphasize the performance of action object detection, but the effect of action object detection on the action recognition performance. From another perspective, ARRs reflect the performance of moving object detection to a certain extent.

The inaccurate detection of action object will lead to the inaccuracy of the size and position of FA so that the recognition performance decreases. For example, the larger FA size causes useless features to be encoded by neurons in V1. To evaluate performance of our attention model and verify the effect of the center localization on action recognition, we implement exhaustive experiments under different conditions: BM obtained by manual and automatic methods, the FA size with fixed value and adaptive value determined by the binary mask of action object. All experiments on Weizmann and KTH datasets are performed four times. The experimental results are shown in Table 4.

**Table 4:** Average Recognition Rates (%) under Field of Attention.

BM	FA Size	Weizmann(ARR/std)	KTH(ARR/std)			
			s1	s2	s3	s4
Automatic	Fixed	98.89/0.53	96.56/1.10	84.10/2.20	89.56/1.10	96.38/1.20
	Adaptive	99.02/0.62	96.77/0.85	91.13/1.15	91.80/1.06	97.10/0.79
Manual	Fixed	99.11/0.52	96.93/0.56	85.12/1.66	92.02/1.45	97.17/1.18
	Adaptive	99.30/0.40	97.47/0.85	91.45/0.96	93.20/0.83	97.37/1.05

According to these results, it is clearly seen that the recognition rates under manual BM are higher than that under automatic BM, and the recognition rates under FA size with adaptive value are higher than that with fixed value. But, the recognition performance on different datasets under automatic BM condition is close to one under manual BM condition except for KTH s3. Even though the bags and clothes of the action object in KTH s3 directly impact on detection of the moving objects, resulting in low performance of action recognition, the recognition rate is still acceptable. It represents that our attention model is effective.

Moreover, it can also be seen from Table 4 that the recognition rate on KTH s2 under FA size with adaptive value is much higher than that with fixed value. The main reason is that the proposed method with automatically adjusting FA size satisfies scale variation of action object, the size of the action objects in KTH s2 changes greatly due to the zoom shots. It indicates that the our model is robust.

### 3 Comparisons with Different Approaches

#### Comparison I-With Bio-inspired Approaches

The purpose of this comparison is to find which bio-inspired approach proposed is more effective. It is more meaningful and fair to make comparison of different approaches on the same dataset. Tables 5 and 6 show the performance comparisons of some bio-inspired approaches on both Weizmann and KTH datasets respectively.

**Table 5:** Comparison with Bio-inspired Approaches on Weizmann Dataset.

Approaches	Setup1(%)	Setup2(%).	Setup3(%)	Years
Ours (CRF+surround)	99.02	98.76	99.36	–
Ours (CRF)	94.65	93.38	95.19	–
Escobar (TD) [5]	–	96.34	98.53	2012
Escobar (SKL) [5]	–	96.48	99.26	2012
Escobar (CRF) [13]	–	90.92	–	2009
Escobar (CRF+surrounds) [13]	–	92.68	–	2009
Jhuang(GrC2 dense features) [4]	–	–	91.10	2007
Jhuang(GrC2 sparse features) [4]	–	–	97.00	2007

**Table 6:** Comparison with Bio-inspired Approaches on KTH Dataset.

Approaches	Setup	s1	s2	s3	s4	avg.
Ours	Setup1	96.77	91.13	91.80	97.10	94.20
	Setup2 (100trails)	96.71	91.06	90.93	97.02	93.93
	Setup3 (5trails)	97.06	91.24	91.87	97.45	94.41
Escobar [5]	Setup2 (100trails)	83.09	–	69.75	83.84	78.89
	Setup3 (5trails)	92.00	–	84.44	92.44	89.63
Ning [31]	Setup1	–	–	–	–	83.79
	Setup2 (100trails)	–	–	–	–	92.31
	Setup3 (5trails)	95.56	87.41	90.66	94.74	92.09
Jhuang [4]	Setup3(dense)	94.30	86.00	85.80	91.00	89.30
	Setup3(sparse)	92.70	86.80	87.50	93.20	90.00

On Weizmann dataset, the best recognition rate is 92.81% under experiment environment Setup 2 by Escobar’s approach [13] which uses the nearest Euclidean distance measure of synchrony motion map with triangular discrimination method, while the best performance of Jhuang’s [4] achieves 97.00% using SVM under experiment environment Setup 3. However, we can draw more conclusions from Table 5. Firstly, no matter what kind of approaches, sparse feature is beneficial to the performance improvement. It is noted that the effective sparse information is obtained by center-surround interaction. Secondly, the comprehensive and reasonable configurations of center-surround interaction can enhance the performance of action recognition. For example, more accurate recognition can achieved by the approach [5] using both isotropic and anisotropic surrounds than the model [59] without these. Finally, our approach obtains the highest recognition performance under different experimental environment even if only isotropic surround interaction is adopted.

From Table 6, it is also seen that the recognition performance of the proposed approach on KTH dataset is superior to others in different experimental setups. For each of four different conditions in KTH dataset, we can obtain the same conclusion. Moreover, our approach is only simulating the processing procedure in V1 cortex without MT cortex, and the number of neurons is less than that of Escobar’s model. The architecture of proposed approach is more simple than that of Escobar’s and Jhuang’s. As a result, our model is easy to implement.

#### Comparison II—Compendium of Results Reported

Due to the lack of a common dataset and standardized evaluation methodology, the development of action recognition algorithms obviously has been limited even if a large number of papers reported good recognition results on individual datasets which contains various human actions. Due to the real difficulties of making such quantitative comparison, the comparison among various different approaches seldom is made cross datasets. Here, in order to ensure consistency and comparability, we simply list some representative studies in terms of the same datasets, and approximate accuracies in Table 7. To some extent, these approaches reflect the latest and best work in human motion or action recognition.

**Table 7:** Comparison of Our approach with Others on KTH Dataset.

Methods	Setup1(%)	Setup2(%).	Setup3(%)	Years
Ours	94.20	93.93	94.41	-
Yuan [61]	95.49	-	-	2013
Zhang&Tao [29]	-	-	93.50	2012
Wang [62]	-	-	94.20	2011
Gilbert [60]	95.70	-	94.50	2011
Kovashka [27]	-	-	94.53	2010
Yuan [63]	-	-	93.30	2009
Leptev [64]	-	-	91.80	2008

In Table 7, we report the experimental results on the KTH dataset. Our experiment setting is consistent with the respective setting in [4], [5], [31], [29], [60], and we train and test the proposed method with Setup1 and Setup3 on the entire dataset. The experimental results of our approach under Setup 2 are also provided. From Table 7, we can see that performance of proposed approach demonstrated here is comparable to others with respect to recognition rates. Moreover, we have also found that recognition rates of our approach are relative stable under different setups in the comparable data set, and the difference between them is not more than 0.5%.

**Fig 16:**
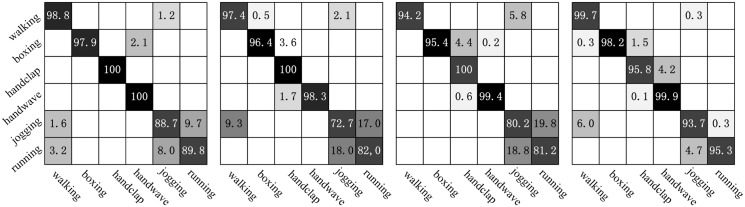
Confusion matrices on KTH dataset. From left to right: s1, s2, s3 and s4.

Fig 16 represents the confusion matrices of the classification on the KTH dataset using our approach. The column of the confusion matrix represents the instances to be classified, while each row represents the corresponding classification results. The main confusion occurs between *jogging* and *running* in four different scenarios. It is a difficult challenge to distinguish the *jogging* and *running* because the two actions performed by some subjects are very similar.

We also use two cross-validation strategies under Setup1 and Setup3 for UCF Sports dataset used in the computer vision. Again, our performance shown in Table 8 is at 90.82% accuracy, and it is better than other contemporary approaches except Wu’ method, which achieves at best 91.3%. These results clearly demonstrate that our approach is a notable new representation for human action in video and capable of robust action recognition in a realistic scenario.

**Table 8:** Comparison of Our approach with Others’ on UFC Sports Dataset.

Methods	Setup1(%)	Setup3(%)	Years
Rodriguez [65]	69.20	-	2008
Varma & Babu [66]	85.20	-	2009
Kovashka [27]	87.30	-	2010
Wu [67]	91.30	-	2011
Wang [62]	88.20	-	2011
Yuan [61]	87.33	-	2013
Ours	90.82	90.96	-

## Discussion and Conclusions

In this paper we propose a bio-inspired model to extract spatiotemporal features from videos for human action recognition. Our model simulates the visual information processing mechanisms of spiking neurons and spiking neural networks composed with them in V1 cortical area. The core of our model is the detection and processing of spatiotemporal information inspired by the visual information perceiving and processing procedure in V1. The dynamic properties of V1 neurons are modeled using 3D Gabor spatiotemporal filter which can detect spatial and temporal information inseparately. To further process spatiotemporal information for effective features extraction and computation of saliency map, we adopt the center surround interactions, inhibition and facilitation based on horizontal connections of neurons in V1. The visual attention model is then integrated into the proposed approach for better action recognition performance. Then the bio-inspired features generated by neuron IF model are encoded with the proposed action code based on the average activity of V1 neurons. Finally the action recognition is finished via a standard classification procedure. In summary, our model has several advantages:

1. Our model only simulates the visual information processing procedure in V1 area, not in MT area of visual cortex. So our architecture is more simple and easier to implement than the other similar models.
2. The spatiotemporal information detected by 3D Gabor, which is more plausible than other approaches, is more effective for action recognition than the spatial and temporal information detected separatively.
3. Salient moving objects are extracted by perceptual grouping and saliency computing, which can blind meaningful spatiotemporal information in the scene but filter the meaningless one.
4. A spiking neuron network is introduced to transform the spatiotemporal information into spikes of neurons, which is more biologically plausible and effective for the representation of spatial and motion information of the action.

Although extensive experimental results have validated the powerful abilities of the proposed model, further evaluation on a larger dataset, with multivaried actions, subjects and scenarios, needs to be carried out. Both shape and motion information derived from actions play important roles in human motion analysis [2]. Fusion of the two information is, thus, preferable for improving the accuracy and reliability. Although there have been some attempts for this problem [30], they usually use the linear combination between shape and motion features to perform recognition. How to extract the integrative features for action recognition still remains challenging.

In addition, the recognition result of our model suggests that the longer subsequences may be more helpful for information detection. However, in many practical applications, it is impossible to recognize action for long time. Most of the application focus on the short sequences. Thus, the feature extraction should be as fast as possible for action recognition.

Finally, surround suppressive motion energy can be computed from video scene based on the definition of the surround suppression weighting function, stimulating biological mechanism of center surround suppression. We can find that the response of texture or noise in one position is inhibited by texture or noise in neighboring regions. Thus, the surround interaction mechanism can decrease the response to texture while not affecting the responses to motion contours, and is robust to the noise. However, as a particular V1 excitatory neuron identified as the target neuron, its surround inhibition properties are known to depend on the stimulus contrast [50], with lower contrasts yielding larger summation RF sizes. To fire the neuron at lower contrast, the neuron has to integrate over a larger area to reach its firing threshold. It requires that the surround size can be automatically adjusted according to local contrast. Therefore, there are still problems to be solved in the model, for instance, the dynamical adjustment of summation RF sizes and further processing of motion information in MT.

## Supporting Information

S1 File The granted permission. (PDF)
