# Supplementary material for: Computational Model of Primary Visual Cortex Combining Visual Attention for Action Recognition
Source: PLoS One. 2015 Jul 1;10(7):e0130569. doi: 10.1371/journal.pone.0130569 (PMC4489578; doi:10.1371/journal.pone.0130569)
Supplement: S1 File — (PDF) [file pone.0130569.s001.PDF]

To the editors of PLOS ONE:

I am the creator of UCSD Anomaly Detection Dataset (see http://www.svcl.ucsd.edu/projects/anomaly/dataset.htm) and hold it's copyright. I hereby permit PLOS ONE to republish UCSD Anomaly Detection Dataset as part of a paper that is covered by a CC BY 3.0 license (http://creativecommons.org/licenses/by/3.0/us/).

Should you need any further information, I would be more than happy to provide . I can be reached by email(wel017@ucsd.edu) and homepage (http://www.svcl.ucsd.edu/~nicolas/).

Best,

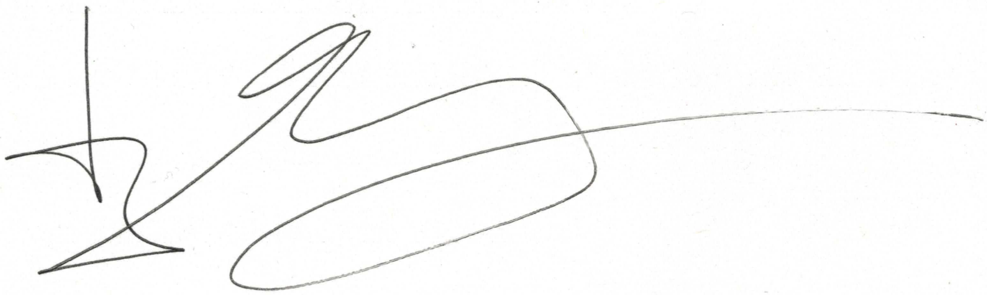

Weixin Li
